# Microglia Drive Peripapillary Vascular Density Reduction in Normal Tension Glaucoma by Regulating the Rpl17/Stat5b/Apoa1 Axis

**DOI:** 10.1002/advs.202507894

**Published:** 2025-09-08

**Authors:** Di Zhang, Sisi Chen, Yanfeng Zhang, Leyi Qiu, Mengxian Du, Wulian Song, Fengyi Guo, Jingyang Zhang, Xinna Liu, Huiping Yuan

**Affiliations:** ^1^ Department of Ophthalmology The Second Affiliated Hospital of Harbin Medical University Harbin 150086 China; ^2^ Key Laboratory of Myocardial Ischemia The Second Affiliated Hospital of Harbin Medical University Harbin 150086 China; ^3^ Future Medical Laboratory The Second Affiliated Hospital of Harbin Medical University Harbin 150086 China

**Keywords:** microglia, neuroprotection, normal tension glaucoma, retinal peripapillary vascular density

## Abstract

Normal tension glaucoma (NTG) is a predominant subset of glaucoma in Asia and is characterized by glaucomatous optic neuropathy in the absence of elevated intraocular pressure. Alterations in retinal blood vessels are reported to be important mechanisms of glaucomatous optic nerve damage. Retinal peripapillary vascular density is assessed in patients with early stage NTG and OPTN (E50K) mutant mice and confirmed a similar reduction in retinal peripapillary vascular density in patients with NTG and model mice. Thus, identifying the mechanisms underlying retinal vascular changes in NTG is crucial for developing effective therapeutic strategies. This study revealed that Rpl17 is upregulated in the retinal microglia of OPTN (E50K) mutant mice. Rpl17 exerts regulatory control over Apoa1 by directly interacting with Stat5b, which causes damage to retinal vascular endothelial cells and leads to a reduction in retinal peripapillary vascular density. Additionally, we identified ellagic acid (EA) as an Apoa1 antagonist that is able to alleviate damage to retinal vascular endothelial cells and increase retinal peripapillary vascular density, which subsequently protected retinal ganglion cells and improved visual function. The work elucidates the vascular mechanism of NTG optic nerve damage and proposes EA as an effective adjuvant therapy strategy.

## Graphical abstract

This study revealed that E50K mutant microglia drive retinal peripapillary vascular density reduction in NTG via the Rpl17/Stat5b/Apoa1 axis and identified Apoa1 as a novel suppressor of retinal vascular endothelial cells. These findings identify a potential therapeutic target for glaucoma and support the clinical potential of EA for protecting RGCs.

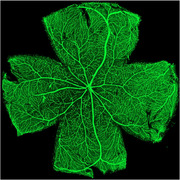

## 1 Introduction

Glaucoma is characterized by specific optic nerve damage and corresponding visual field loss, and it is the primary cause of irreversible blindness worldwide.^[^ ^1^ ^]^ Normal tension glaucoma (NTG) is the most prevalent subtype in Asia.^[^ ^2^ ^]^ It is a multifactorial optic neuropathy characterized by progressive retinal ganglion cells (RGCs) death and visual field loss without high intraocular pressure (IOP).^[^ ^2^, ^3^ ^]^ Patients with NTG can still have progressive visual impairment after IOP‐lowering treatment.^[^ ^4^, ^5^ ^]^ Therefore, to elucidate the mechanism of optic nerve damage in NTG and develop effective treatments, we established OPTN (E50K) mutant mice as an NTG model using CRISPR/Cas9 gene‐editing technology and successfully demonstrated age‐related glaucomatous features in E50K mice.^[^ ^2^, ^6^, ^7^, ^8^ ^]^

Emerging evidence shows that retinal vascular impairment and insufficient blood supply play key roles in the development of glaucomatous optic neuropathy.^[^ ^9^, ^10^, ^11^ ^]^ Many glaucoma patients exhibit neurovascular dysfunction, including decreased blood flow and vascular density in the retina and optic nerve head (ONH), reduced vascular caliber, and capillary defects.^[^ ^12^, ^13^, ^14^, ^15^ ^]^ In particular, in patients with NTG, where IOP is not elevated, vascular factors may play a more significant role. Moreover, reduced blood flow in the ONH and surrounding regions is considered an early risk factor for disease progression.^[^ ^15^, ^16^, ^17^ ^]^ Nonetheless, the mechanisms underlying abnormal vascular function in glaucoma patients and the impact on RGCs damage remain unclear.

The retinal neurovascular unit (NVU) is defined as the community of RGCs and surrounding cells, including vascular endothelial cells, microglia and astrocytes. It is now clear that the complex signaling pathways between the diverse types of cells in the NVU lead to alterations in blood flow and vascular barriers via neurovascular responses, which are important for providing nutrition and oxygen, as well as clearing potentially toxic byproducts in the retina.^[^ ^14^, ^15^, ^18^, ^19^, ^20^ ^]^ Correspondingly, dysfunction of NVU components and abnormal vascular responses are important factors in diabetes, Alzheimer's disease and other conditions.^[^ ^21^, ^22^, ^23^, ^24^ ^]^ We also observed reduced retinal peripapillary vascular density in both early‐stage patients with NTG and NTG model mice. Therefore, exploring the underlying interactions among these cells in the NVU in glaucomatous optic nerve damage is crucial, especially for discovering new therapeutic strategies. We found that microglia play an important role in NTG optic nerve damage and that the signaling cascades between microglia and vascular endothelial cells regulate neurovascular responses, affecting RGCs viability and visual function.

In this study, we report that high levels of apolipoprotein A1 (Apoa1), which is regulated by ribosomal protein L17 (Rpl17) in the retinal microglia of OPTN (E50K) mutant mice, are responsible for the apoptosis of vascular endothelial cells and the reduction in retinal peripapillary vascular density in NTG, which further augments RGCs damage. Promisingly, inhibiting Apoa1 improved RGCs survival and visual function in E50K mice. Our findings elucidate the function of neurovascular cells and their underlying interactions during NTG optic nerve damage and identify a promising therapeutic target.

## 2 Results

### 2.1 Retinal Peripapillary Vascular Density is Reduced in Normal‐Tension Glaucoma

**Figure 1:**
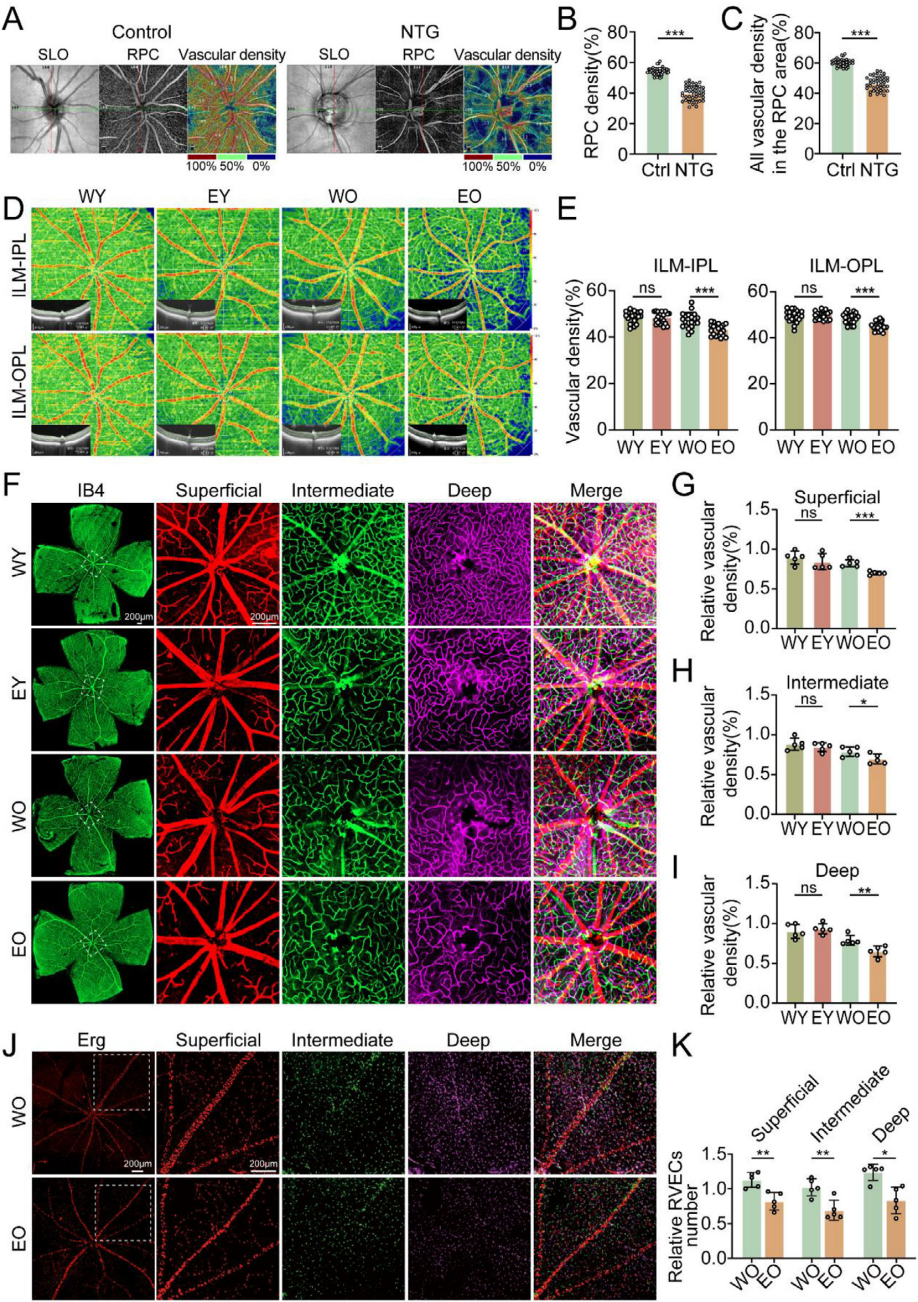
Retinal peripapillary vascular density is reduced in patients with NTG and models. A) OCTA results of 40 patients with NTG and 40 healthy controls. B) RPC density statistical graph of the OCTA results. C) All vascular density data in the RPC area were statistically analyzed via OCTA. D) OCTA results of WY, EY, WO and EO mice (n = 20). E) Statistical graph of the ILM‐IPL results (left panel). Statistical graph of the ILM‐OPL results in the right panel. F) IB4 staining of the retinal vasculature of WY, EY, WO and EO mice (n = 5); scale bar = 200 µm. G) Statistical graph of superficial retinal layer peripapillary vascular density results. H) Statistical graph of intermediate retinal layer peripapillary vascular density results. I) Statistical graph of deep retinal layer peripapillary vascular density results. J) Erg‐stained retinal vessels of WO and EO mice (n = 5); scale bar = 200 µm. K) Statistical graph of the Erg staining results. The data were analyzed via the Welch t test (B, C), Student's t test (E, G, H, and K) or the Mann‒Whitney U test (I). Significant results are presented as ^*^ *P* < 0.05, ^**^ *P* < 0.01, and ^***^ *P* < 0.001.

First, we conducted an analysis of retinal peripapillary vascular density in 40 patients in the early stage of NTG and 40 healthy controls by optical coherence tomography angiography (OCTA) and found a significant decrease in both the radial peripapillary capillary (RPC) density and all vascular density in the RPC area in the early stage in patients with NTG, which is consistent with the findings of previous clinical studies (**Figure** **1**A–C; Figures S1 and S2, Supporting Information). We subsequently calculated the retinal peripapillary vascular density in old OPTN (E50K) mice (EO) with a typical NTG phenotype and in age‐matched wild‐type control (WO) mice via small‐animal OCTA imaging. Similarly, the peripapillary vascular density in the internal limiting membrane (ILM) to the inner plexiform layer (IPL) (ILM‐IPL) and the ILM to the outer plexiform layer (OPL) (ILM‐OPL) of the retina was significantly lower in the EO group than in the WO group. In addition, no statistically significant difference in peripapillary vascular density in the ILM‐IPL or ILM‐OPL layers of the retina was detected between young E50K mice (EY) and age‐matched WT controls (WY) (Figure 1D,E; Figures S3–S6, Supporting Information). Isolectin B4 (IB4) was used to visualize the retinal vasculature, and the results further confirmed that peripapillary vascular density in the superficial, intermediate and deep retinal layers did not change in the EY group but was significantly reduced in the EO group (Figure 1F–I). Immunofluorescence staining also revealed a significant reduction in the number of retinal vascular endothelial cells (RVECs) in the superficial, intermediate and deep retinal layers of the EO mice compared with those in the WO mice (Figure 1J,K). We used flow cytometry and cell sorting to isolate RVECs from EO and WO mice (Figure S7A, Supporting Information). Increased RVECs apoptosis was detected in EO mice, which was confirmed by increased expression of Bax and cleaved caspase 3 and decreased expression of Bcl2 (Figure S7B–E, Supporting Information).

Overall, these results provide evidence that the OPTN (E50K) mutation induces RVECs apoptosis, leading to a reduction in retinal peripapillary vascular density.

### 2.2 Rpl17 Overexpression in OPTN (E50K) Mutant Microglia Represses the Viability of Vascular Endothelial Cells

**Figure 2:**
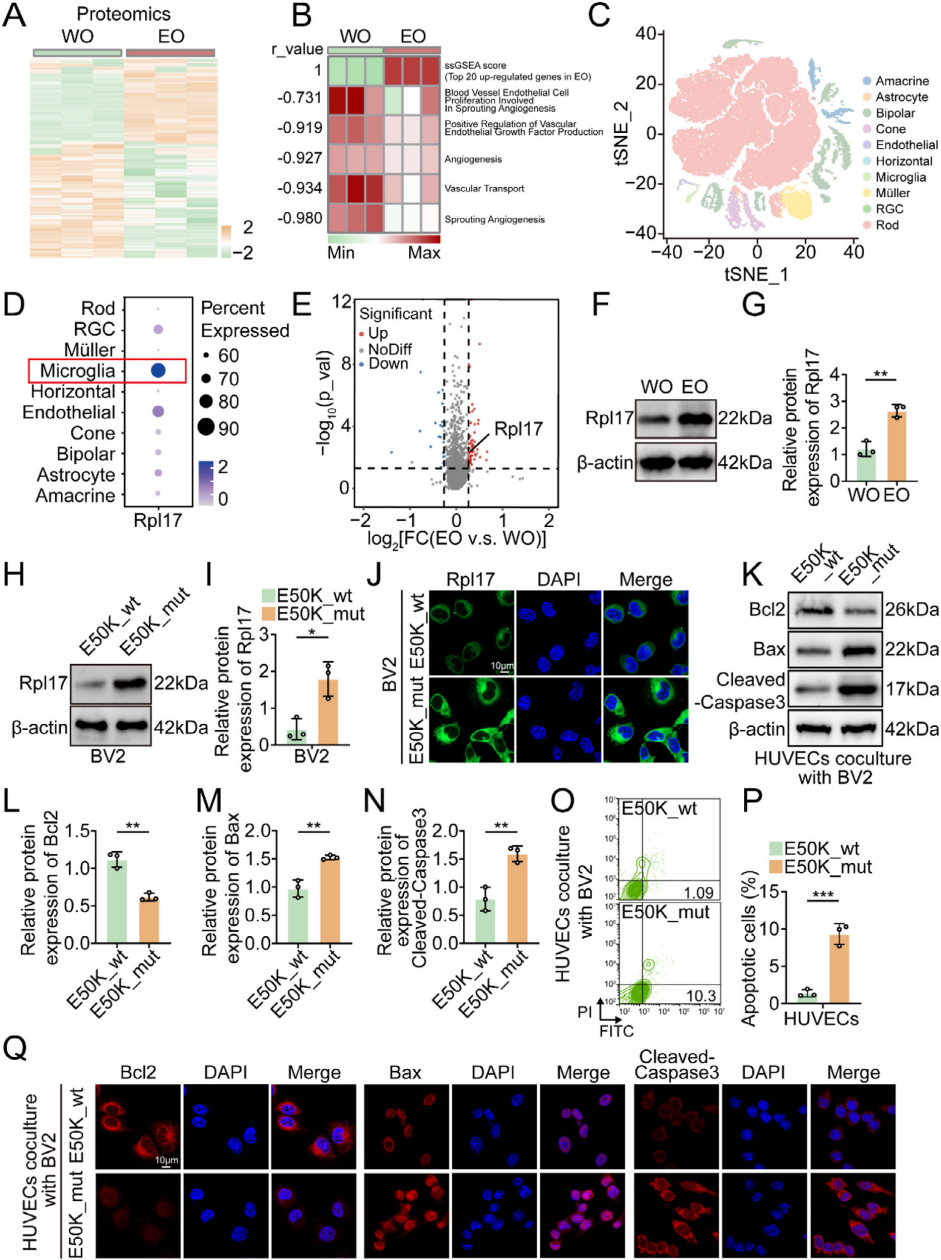
Rpl17 overexpression in OPTN (E50K) mutant microglia induces vascular endothelial cell apoptosis. A) Differentially expressed proteins in the retinas of WO and EO mice. B) The top 20 upregulated proteins associated with the functional gene sets of EO mice are shown in the heatmap. C) t‐SNE plot showing identified cell clusters in the retina. D) Bubble plots illustrating the expression levels of Rpl17 in identified cell clusters. E) Volcano plot showing differentially expressed genes in the microglial cluster (EO vs. WO). F) Western blot analysis of Rpl17 levels in the retinas of WO and EO mice (n = 3). G) Statistical analysis of the western blot data. H) Western blot analysis of Rpl17 levels in E50K_wt and E50K_mut BV2 cells (n = 3). I) Statistical analysis of the western blot data. J) IF analysis of Rpl17 levels in E50K_wt and E50K_mut BV2 cells. The nuclei were stained with DAPI; scale bar = 10 µm. K) Western blot analysis of the indicated proteins in HUVECs cocultured with E50K_wt or E50K_mut BV2 cells for 24 h (n = 3). L–N) Statistical analysis of the western blot data. O) Flow cytometry analysis revealing the degree of apoptosis in HUVECs cocultured with E50K_wt or E50K_mut BV2 cells for 24 h (n = 3). P) Statistical analysis of the flow cytometry data. Q) IF analysis of the indicated proteins in HUVECs cocultured with E50K_wt or E50K_mut BV2 cells for 24 h. The nuclei were stained with DAPI; scale bar = 10 µm. The data were analyzed using the Wilcoxon test (E) and Student's t test (G, I, L, M, N and P). Significant results are presented as ^*^ *P* < 0.05, ^**^ *P* < 0.01, and ^***^ *P* < 0.001.

To investigate the potential mechanisms underlying the effects of the E50K mutation on the viability of vascular endothelial cells, we performed proteomic analysis and single‐cell RNA sequencing on the retinas of EO and WO mice. We identified 467 upregulated proteins and 806 downregulated proteins (|FC| > 1, *p* < 0.05) in the EO retina from the proteomic results (**Figure** **2**A). Single‐sample gene set enrichment analysis (ssGSEA) revealed that the top 20 upregulated proteins were enriched in the KEGG pathways associated with the inhibition of vascular development and function (Figure 2B). Among the upregulated proteins, Rpl17 has been reported to be involved in vascular development and function. In addition, Rpl17 is preferentially expressed in microglia rather than other retinal cell types according to our single‐cell RNA sequencing results (Figure 2C,D), especially in the microglia of the EO retina (Figure 2E). We also confirmed that Rpl17 expression was greater in EO retinas than in WO retinas. (Figure 2F,G). After depletion of endogenous OPTN, BV2 cells were reconstituted with OPTN E50K (E50K_mut) as an in vitro E50K mutant microglial model, and WT OPTN (E50K_wt) was used as a control (Figure S7F, Supporting Information). Similarly, Rpl17 expression levels were markedly higher in E50K_mut BV2 cells than in E50K_wt BV2 cells (Figure 2H–J).

To validate the effect of E50K‐mutant microglia on vascular endothelial cell function, we cocultured E50K_wt BV2 cells and E50K_mut BV2 cells with human umbilical vein endothelial cells (HUVECs) for 24 h. E50K_mut BV2 cells prominently induced apoptotic characteristics in HUVECs (Figure 2K–P). Immunofluorescence further confirmed that HUVECs cocultured with E50K_mut BV2 cells presented increased expression of apoptotic proteins and decreased expression of antiapoptotic proteins (Figure 2Q). These data reveal that E50K‐mutant microglia have inhibitory effects on vascular endothelial cell viability.

### 2.3 Elevated Rpl17 in OPTN (E50K) Mutant Microglia Induces the Modulation of Apoa1 Levels

**Figure 3:**
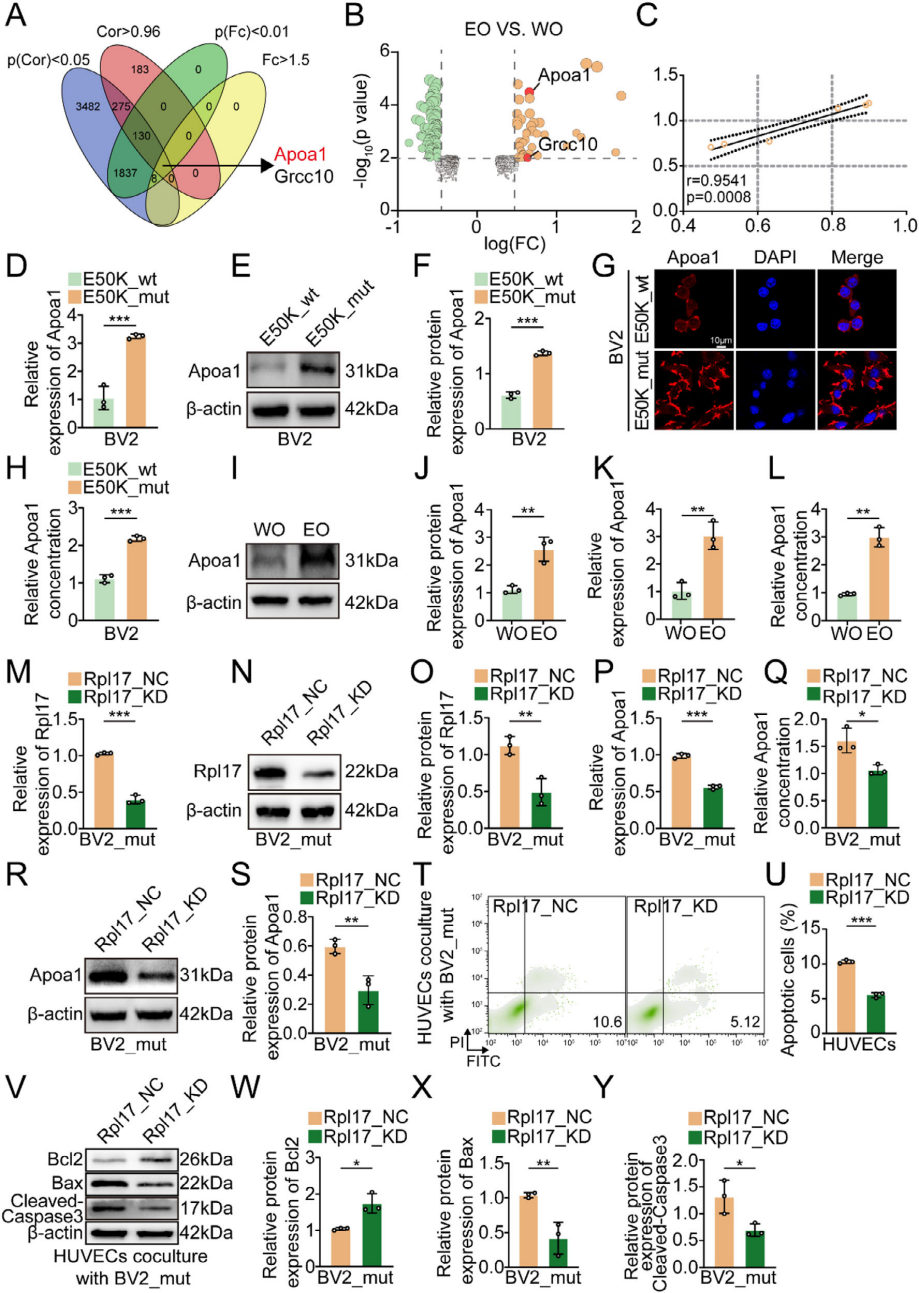
Apoa1 regulated by Rpl17 is highly expressed in E50K_mut microglia and is required for vascular endothelial cell apoptosis. A) Venn diagram illustrating the strategy for screening candidate proteins regulated by Rpl17. B) The Apoa1 and Grcc10 expression levels are presented in a volcano plot. C) Correlation between Apoa1 expression and Rpl17 expression. D) qRT‒PCR analysis of Apoa1 expression in E50K_wt and E50K_mut BV2 cells (n = 3). E. Western blot analysis of Apoa1 levels in E50K_wt and E50K_mut BV2 cells (n = 3). F) Statistical analysis of the western blot data. G) IF analysis of Apoa1 levels in E50K_wt and E50K_mut BV2 cells. The nuclei were stained with DAPI; scale bar = 10 µm. H) ELISA analysis of Apoa1 in culture medium from the indicated BV2 cells (E50K_wt/E50K_mut) (n = 3). I) Western blot analysis of Apoa1 levels in the retinas of WO and EO mice (n = 3). J) Statistical analysis of the western blot data. K) qRT‒PCR analysis of the expression of Apoa1 in the retinas of WO and EO mice (n = 3). L) ELISA analysis of Apoa1 in the retinas of WO and EO mice (n = 3). M) qRT‒PCR analysis of Rpl17 expression after Rpl17 knockdown in E50K_mut BV2 cells (n = 3). N) Western blot analysis of Rpl17 levels after Rpl17 knockdown in E50K_mut BV2 cells (n = 3). O. Statistical analysis of the western blot data. P. qRT‒PCR analysis of Apoa1 expression after Rpl17 knockdown in E50K_mut BV2 cells (n = 3). Q) ELISA analysis of Apoa1 after Rpl17 knockdown in E50K_mut BV2 cells (n = 3). R) Western blot analysis of Apoa1 levels after Rpl17 knockdown in E50K_mut BV2 cells (n = 3). S) Statistical analysis of the western blot data. T) Flow cytometry analysis revealing the degree of apoptosis in HUVECs cocultured with E50K_mut BV2 cells with Rpl17 knockdown (n = 3). U) Statistical analysis of the flow cytometry data. V) Western blot analysis of the indicated protein levels in HUVECs cocultured with E50K_mut BV2 cells with Rpl17 knockdown (n = 3). W–Y) Statistical analysis of the western blot data. Simple linear regression analysis was conducted, and the correlation coefficient (r) was calculated via a goodness‐of‐fit test (C). The data were analyzed using the Student's t test (D, F, H, J, K, M, O, P, Q, S, U, W, X and Y) or Welch's t test (L). Significant results are presented as ^*^ *P* < 0.05, ^**^ *P* < 0.01, and ^***^ *P* < 0.001.

To elucidate the molecular mechanisms by which elevated Rpl17 in OPTN (E50K) mutant microglia causes vascular abnormalities, a correlative analysis was performed between Rpl17 and differentially expressed proteins identified through proteomic profiling of retinal tissues from EO and WO mice. As shown in **Figure** **3**A, Apoa1 and Grcc10 were highly abundant in the EO retina. Compared with Grcc10, Apoa1 tended toward higher expression levels with greater statistical significance and was significantly correlated with Rpl17 (Figure 3B,C). High Apoa1 expression and secretion levels were clearly observed in both E50K_mut BV2 cells and EO retinas compared with those in the control groups (Figure 3D–L).

To further define the relationship between Rpl17 and Apoa1, we monitored the mRNA and protein expression levels of Apoa1 in E50K_mut BV2 cells transfected with Rpl17 knockdown lentivirus (Rpl17_KD). The Apoa1 expression levels and secretion levels were significantly decreased following Rpl17 knockdown (Figure 3M–S). The apoptotic characteristics of HUVECs induced by E50K_mut BV2 cells were reversed after Rpl17 depletion (Figure 3T–Y). Collectively, our findings provide evidence that E50K‐mutant microglia lead to the apoptosis of vascular endothelial cells via the modulation of Apoa1 expression, which is induced by Rpl17 in microglia.

### 2.4 Rpl17 Modulates Apoa1 Expression via Direct Physical Interaction with Stat5b

**Figure 4:**
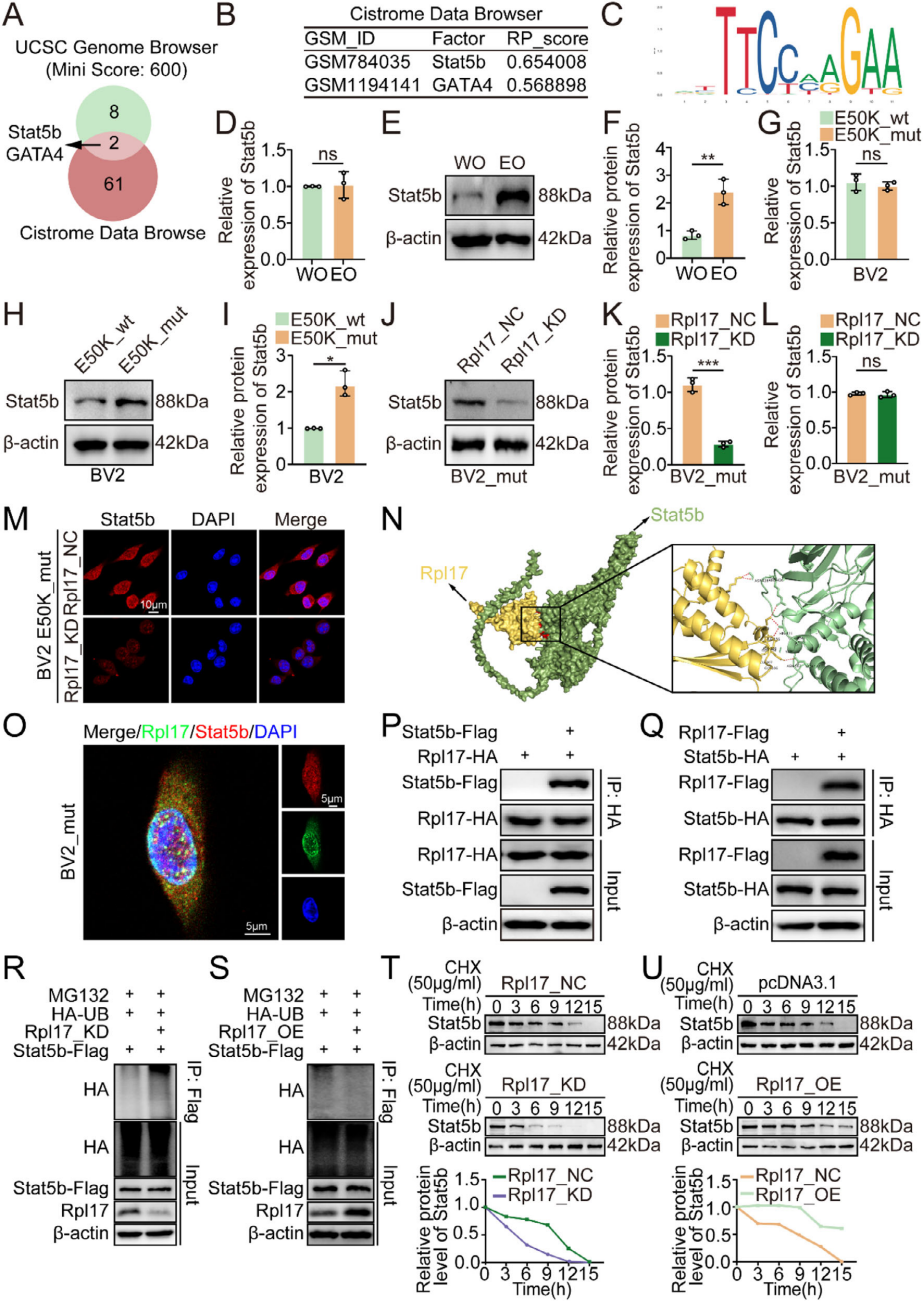
Rpl17 prevents the degradation of Stat5b. A) Venn diagram illustrating the strategy for screening the candidate transcription factors of Apoa1. B) ChIP‐seq binding results searched using the Cistrome Data Browser. C) Predicted Stat5b‐binding sites in the promoter region of Apoa1. D) qRT‒PCR analysis of Stat5b expression in the retinas of WO and EO mice (n = 3). E) Western blot analysis of Stat5b levels in the retinas of WO and EO mice (n = 3). F) Statistical analysis of the western blot data. G) qRT‒PCR analysis of Stat5b expression in E50K_wt and E50K_mut BV2 cells (n = 3). H) Western blot analysis of Stat5b levels in E50K_wt and E50K_mut BV2 cells (n = 3). I) Statistical analysis of the western blot data. J) Western blot analysis of Stat5b levels after Rpl17 knockdown in E50K_mut BV2 cells (n = 3). K) Statistical analysis of the western blot data. L) qRT‒PCR analysis of Stat5b expression after Rpl17 knockdown in E50K_mut BV2 cells (n = 3). M) IF analysis of Stat5b levels after Rpl17 knockdown in E50K_mut BV2 cells. The nuclei were stained with DAPI; scale bar = 10 µm. N) Graphical representation of 3D structures of the docking models of Rpl17 with Stat5b and zoomed‐in images showing the interaction interface of amino acids in the binding site. O) E50K_mut BV2 cells were immunostained for Rpl17 (in green) and Stat5b (in red); yellow in the merged magnified images indicates colocalization. The nuclei were stained with DAPI; scale bar = 5 µm. P,Q) Immunoprecipitation was performed using anti‐HA agarose on lysates derived from BV2 cells exogenously expressing Flag‐tagged Stat5b and HA‐tagged Rpl17 (P) or Flag‐tagged Rpl17 and HA‐tagged Stat5b (Q). R) BV2 cells were infected with the indicated constructs. At 72 h postinfection, the cells were collected for western blot analysis after being treated with MG132 for 8 h. S) BV2 cells were infected with the indicated constructs. At 72 h postinfection, the cells were collected for western blot analysis after being treated with MG132 for 8 h. T) BV2 cells were infected with Rpl17_KD lentivirus. After 72 h, the cells were treated with cycloheximide (CHX), and the cells were collected for western blot analysis at different time points. U) BV2 cells were transfected with the indicated plasmids. After 72 h, the cells were treated with CHX, and the cells were collected for western blot analysis at different time points. The data were analyzed using Welch's t test (D and I) or Student's t test (F, G, K and L). Significant results are presented as ^*^ *P* < 0.05, ^**^ *P* < 0.01, and ^***^ *P* < 0.001.

To gain deeper insight into the mechanism underlying Rpl17‐mediated upregulation of Apoa1 expression, we used the UCSC Genome Browser and Cistrome Data Browser databases for analysis of Apoa1 transcription factors (**Figure** **4**A). We observed that Stat5b and GATA4 share the same transcriptional orientation as Apoa1 does, while Stat5b is predicted to have a strong binding affinity with the Apoa1 promoter (Figure 4B). We also performed bioinformatics analysis of the promoter region of the Apoa1 gene and predicted DNA binding elements (DBEs) for Stat5b binding sites in the JASPAR database (Figure 4C). Interestingly, the EO group presented an increase in Stat5b protein levels without significant changes in Stat5b mRNA expression (Figure 4D–F). Similarly, Stat5b protein levels were greater in E50K_mut BV2 cells than in E50K_wt BV2 cells without significant changes in Stat5b mRNA expression (Figure 4G–I). Moreover, we found that Rpl17 knockdown led to a decrease in Stat5b protein levels without significantly affecting Stat5b mRNA expression (Figure 4J–M).

To determine the interfacial interaction of sequences between Rpl17 and Stat5b, computational structural modeling was performed, and the results were confirmed through a protein‒protein molecular docking experiment (Figure 4N). Further immunofluorescence‐based coexpression assays revealed that the green fluorescence representing Rpl17 overlapped with the red fluorescence representing Stat5b, which also suggested strong colocalization between Rpl17 and Stat5b (Figure 4O). Exogenously expressed Flag‐tagged Stat5b or Rpl17 were eluted with HA‐tagged Rpl17 or Stat5b, respectively (Figure 4P,Q). Furthermore, Rpl17 knockdown increased Stat5b ubiquitination levels and shortened the half‐life of Stat5b (Figure 4R,T). Conversely, Rpl17 overexpression reduced Stat5b ubiquitination levels and prolonged the half‐life of Stat5b (Figure 4S,U). Together, our results demonstrate that Rpl17 governs Stat5b stability and indirectly modulates Apoa1 transcription.

### 2.5 Ellagic Acid (EA) is a Potential Apoa1 Inhibitor with Beneficial Effects for the Reduction of Retinal Peripapillary Vascular Density

**Figure 5:**
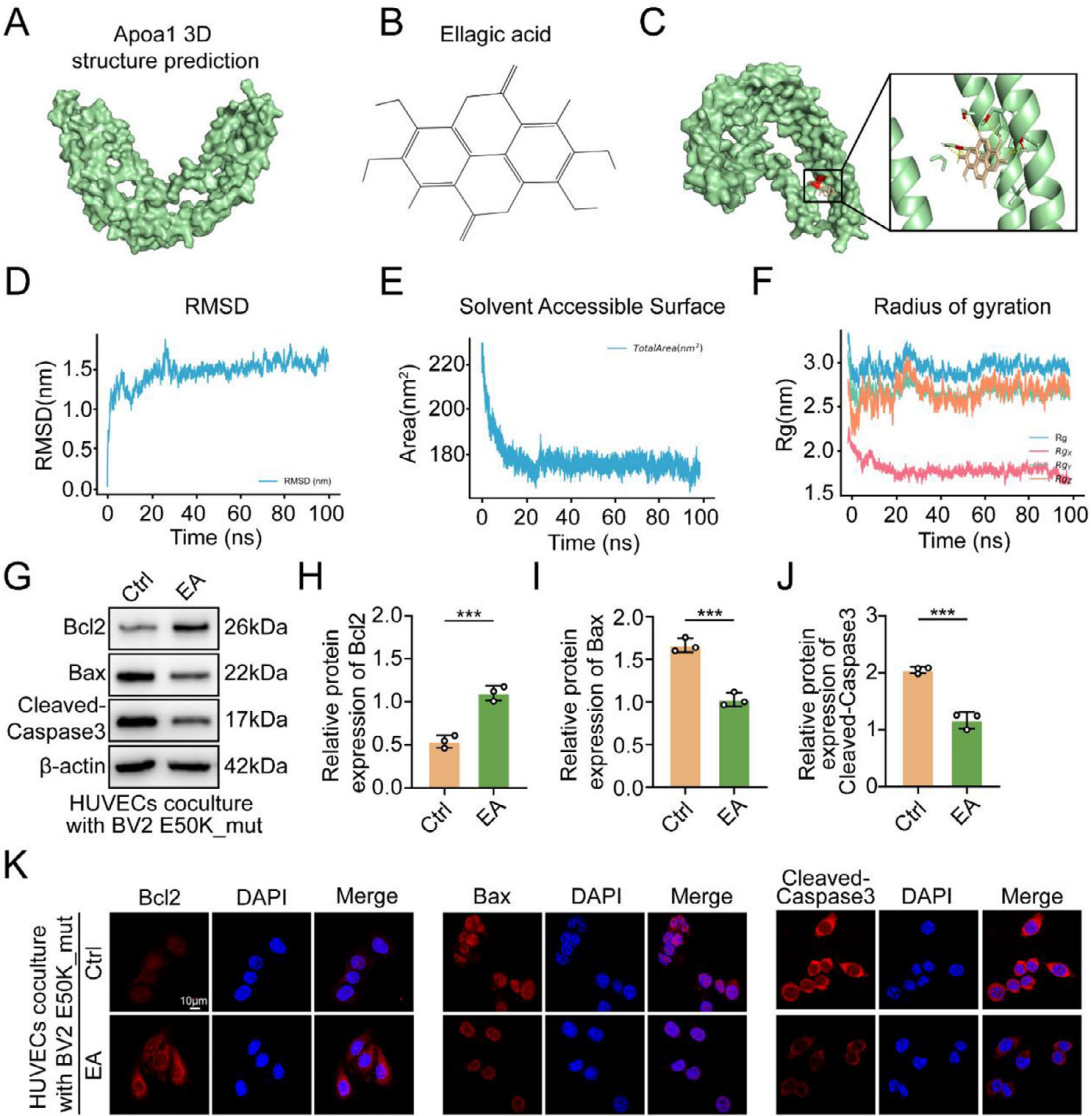
Protective effects of the Apoa1 inhibitor EA on retinal peripapillary vascular density. A) Protein structure prediction of Apoa1. B) Chemical structure of EA. C) Predicted model of EA binding to Apoa1, as shown by computational docking. D) Root mean square deviation (RMSD). E) Area per residue over the trajectory/solvent accessible surface area (SASA). F) Radius of gyration (total and around axes) (Rg). G) Western blot analysis of the indicated proteins in HUVECs cocultured with E50K_mut BV2 cells treated with or without EA (15 µM) (n = 3). H–J) Statistical analysis of the western blot data. K) IF analysis of the indicated proteins in HUVECs cocultured with E50K_mut BV2 cells treated without or with EA (15 µM). The data were analyzed using Student's t test (H, I and J). Significant results are presented as ^*^ *P* < 0.05, ^**^ *P* < 0.01, and ^***^ *P* < 0.001.

Our previous results indicated that targeting the Rpl17/Stat5b/Apoa1 axis could be a potential neuroprotective strategy for NTG. Since Apoa1 is an extracellularly secreted protein that induces apoptosis in RVECs, we investigated whether small‐molecule inhibitors could suppress Apoa1 activity and thereby reverse the reduction in retinal peripapillary vascular density observed in NTG. By examining the active sites, we found that EA formed hydrogen bonds in the hydrophobic pockets of Apoa1 (**Figure** **5**A–C). Molecular dynamics simulation results, including RMSD, SASA, and Rg values, indicated stable and favorable interactions between EA and Apoa1 (Figure 5D–F). EA alleviated the apoptotic characteristics of HUVECs induced by E50K_mut BV2 cells (Figure 5G–J). Immunofluorescence further confirmed that EA reduced apoptosis in HUVECs cocultured with E50K_mut BV2 cells, as evidenced by the decreased expression of apoptotic proteins and increased expression of antiapoptotic proteins (Figure 5K).

**Figure 6:**
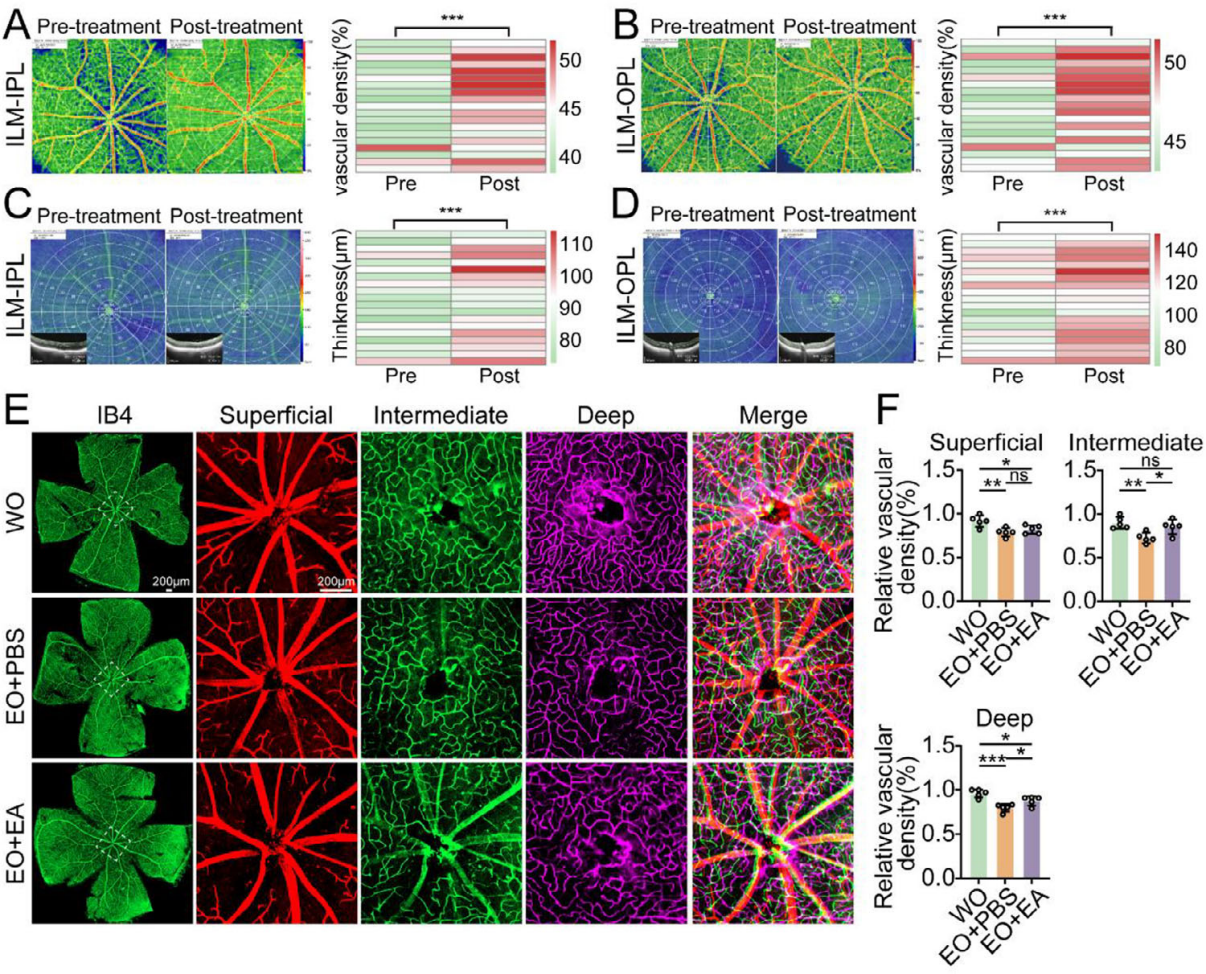
EA mitigates the reduction in retinal peripapillary vascular density. A) Retinal peripapillary vascular density of the ILM‐IPL results of EO mice following EA treatment (75 mg·kg^−1^·day^−1^) (n = 19). B) Retinal peripapillary vascular density of ILM‐OPL results of EO mice following EA treatment (75 mg·kg^−1^·day^−1^) (n = 19). C. Retinal thickness of the ILM‐IPL results of EO mice following EA treatment (75 mg·kg^−1^·day^−1^) (n = 19). D. Retinal thickness of the ILM‐OPL results of EO mice following EA treatment (75 mg·kg^−1^·day^−1^) (n = 19). E. IB4 staining of the retinal vasculature of WO, EO, and EO treated with EA (75 mg·kg^−1^·day^−1^) mice (n = 5); scale bar = 200 µm. F. Statistical graph of the retinal peripapillary vascular density results. The data were analyzed using Student's t test (A, B, C, D and F). Significant results are presented as ^*^ *P* < 0.05, ^**^ *P* < 0.01, and ^***^ *P* < 0.001.

**Figure 7:**
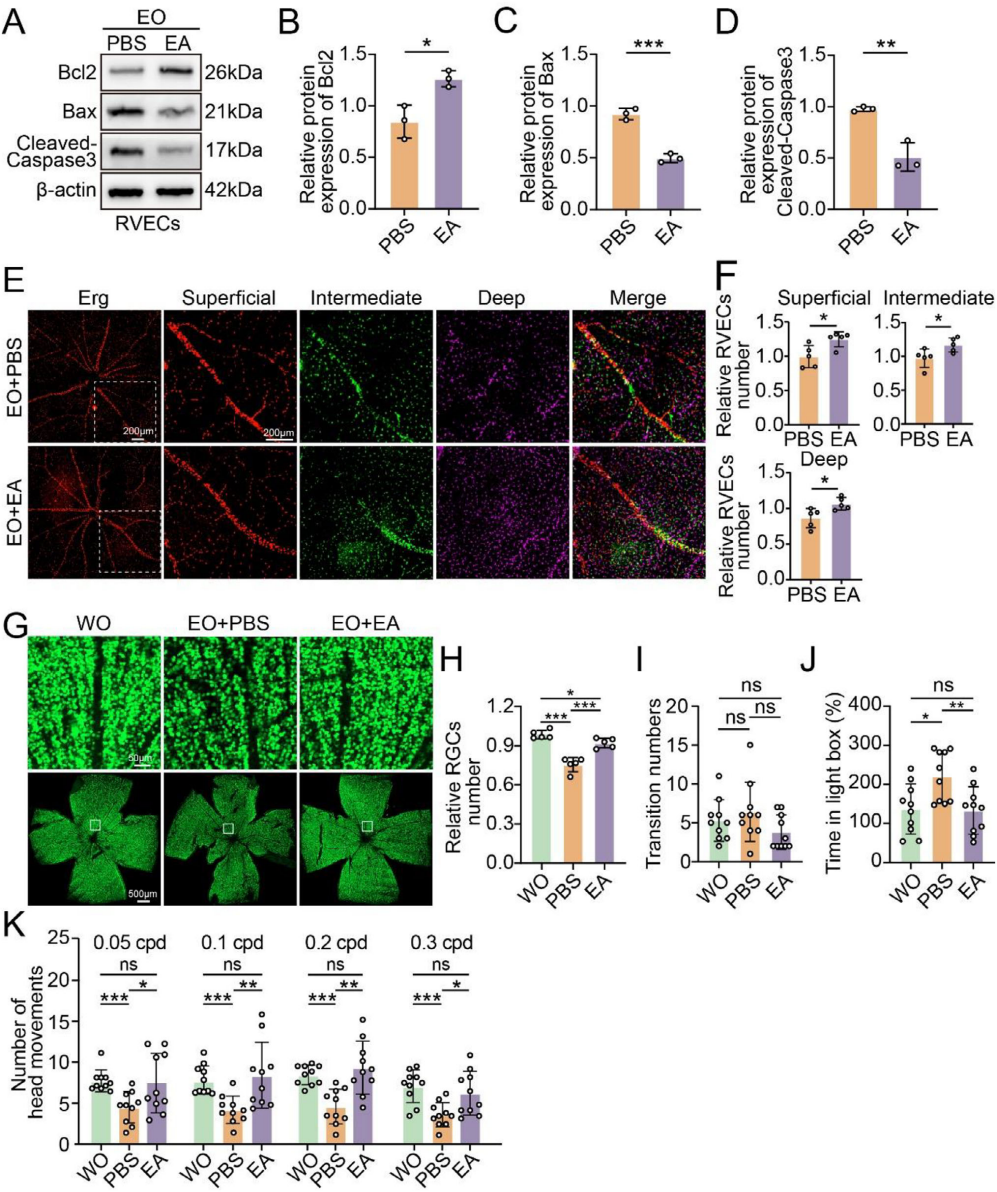
EA alleviates the apoptosis of retinal vascular endothelial cells. A) Western blot analysis of the indicated proteins in the RVECs of EO mice treated with or without EA (75 mg·kg^−1^·day^−1^) (n = 3). B–D) Statistical analysis of the western blot data. E) Erg‐stained retinal vessels of EO mice treated with or without EA (75 mg·kg^−1^·day^−1^) (n = 5); scale bar = 200 µm. F) Statistical analysis of the endothelial cell count results (n = 5). G) Immunofluorescence staining for the RGCs marker NeuN was performed on the retinas of EO mice treated with or without EA (75 mg·kg^−1^·day^−1^) (n = 5); scale bar = 50 µm. H) Statistical analysis of the RGCs count results. I,J) L/D‐T test (n = 10). K) OMR test (n = 10). The data were analyzed using Student's t test (B, C, D F, H and K) or the Mann‒Whitney U test (I and J). Significant results are presented as ^*^ *P* < 0.05, ^**^ *P* < 0.01, and ^***^ *P* < 0.001.

To examine the ability of EA to alleviate RVECs apoptosis in vivo, EO mice were treated with EA for 4 weeks via oral administration.^[^ ^25^ ^]^ These EO mice were inspected via OCTA both before and after EA treatment to assess alterations in retinal peripapillary vascular density. The OCTA results revealed that the peripapillary vascular density in both the ILM to IPL and the ILM to OPL significantly increased after EA treatment (**Figure** **6**A,B; Figures S8 and S9, Supporting Information). Moreover, the corresponding retinal thickness also significantly increased (Figure 6C,D; Figures S8 and S9, Supporting Information). IB4 staining revealed that EA completely reversed the reduction in intermediate peripapillary vessels in the EO group and partially reversed the reduction in deep peripapillary vessels and superficial peripapillary vessels (Figure 6E,F). Additionally, RVECs were isolated by flow sorting, and protein was extracted to evaluate the expression of apoptosis‐related proteins. The results revealed that RVECs apoptosis was significantly reversed following EA treatment (**Figure** **7**A–D).

Immunofluorescence staining revealed a significant reversal of the reduction in the number of RVECs in the superficial, intermediate, and deep retinal layers of EO mice following EA treatment (Figure 7E,F). We also found a significant improvement in the reduction in the number of RGCs in the EO mice following EA treatment (Figure 7G,H). In the light/dark transition (L/D‐T) test, the EA‐treated mice spent significantly less time in the light compartment than did the EO group, indicating a significant improvement in visual function. However, there was no statistically significant difference in the number of movements among the three groups, indicating that the exploratory behavior and motor ability of the mice were similar. (Figure 7I,J). Similarly, the optomotor response (OMR) test revealed that after EA treatment, the EO mice presented significant increases in head movements at 0.05, 0.1, 0.2, and 0.3 cpd compared with those of the untreated EO mice, with no significant difference from those of the WO mice (Figure 7K). The safety of EA therapy was also confirmed, with no pathological changes observed in the visceral organs, and no abnormalities in retinal vessels detected via fundus fluorescein angiography after EA treatment (Figure S10A,B, Supporting Information). Overall, these results provide evidence that EA is a potential Apoa1 inhibitor with protective effects in mitigating the reduction in retinal peripapillary vascular density, thereby restoring visual function in NTG mice.

## 3 Discussion

Glaucoma is a group of neurodegenerative diseases characterized by the progressive loss of RGCs. However, the underlying molecular mechanism still requires further investigation. Many studies have confirmed that RGCs, as the main neurons for conveying visual information from the retina to the brain, have an extremely high metabolic demand to sustain their activity.^[^ ^26^, ^27^ ^]^ Therefore, nerve vascular systems that maintain the delivery of oxygen and nutrients are truly important, especially the RPC plexus for the ONH, the primary site of RGCs injury.^[^ ^28^, ^29^, ^30^ ^]^ Insufficient blood supply and vascular deficits in the ONH fail to meet the metabolic demand of RGCs and propel their eventual death. Several cross‐sectional studies have shown that glaucoma patients with both high and normal IOP have significant blood flow alterations in the ONH, which is correlated with disease progression.^[^ ^31^, ^32^, ^33^, ^34^, ^35^ ^]^ Consistent with previous findings, we also observed a significant reduction in retinal peripapillary vascular density in patients in the early stage of NTG.^[^ ^15^, ^36^, ^37^, ^38^, ^39^ ^]^ Thus, identifying the regulatory mechanism of the neurovascular response in the ONH is a key focus in glaucomatous pathogenesis and RGCs protection.

These studies indicate that high IOP is not the sole cause for these vascular changes. Our study revealed an increase in RVECs apoptosis corresponding to a marked reduction in retinal peripapillary vascular density in NTG mice. Vascular endothelial cells, as integral components of the vascular wall, are responsible for maintaining vascular integrity, regulating perfusion, and promoting angiogenesis.^[^ ^40^, ^41^, ^42^ ^]^ RVECs apoptosis and death reportedly lead to narrow vascular calibres and vascular density alterations.^[^ ^43^, ^44^, ^45^ ^]^ Thus, further exploration of the impacts of vascular endothelial cell dysfunction on glaucomatous neurodegeneration is warranted.

Microglia in the NVU, which are resident immune cells in the retinal microenvironment, are sensitive to subtle changes in their surroundings and maintain tissue homeostasis by communicating with other retinal cells.^[^ ^46^, ^47^, ^48^, ^49^ ^]^ Microglia have been reported to play a pivotal role in retinal vascular development by interacting with endothelial cells to change vascular shape via paracrine effects.^[^ ^50^, ^51^, ^52^ ^]^ However, the molecular mechanisms by which microglia and endothelial cells interact with each other to coordinate neurovascular responses to fulfill RGCs requirements and maintain retinal homeostasis remain unknown. In this study, our results provide evidence that the overexpression of Rpl17 in the microglia of OPTN (E50K) mutant mice leads to increased Apoa1 secretion via the stabilization of Stat5b, which promotes RVECs apoptosis and ultimately results in decreased retinal peripapillary vascular density. Our research suggests that Rpl17 and Apoa1 may play significant roles in the microglia‐endothelial interaction.

Rpl17, a ribosomal protein, has been reported to function as an inhibitor of vascular smooth muscle growth and regulate vascular function via an extraribosomal function in cardiovascular diseases.^[^ ^53^ ^]^ Apoa1 is a principal protein moiety in high‐density lipoprotein, which is atheroprotective. Generally, Apoa1 levels are associated with lower CVD risk. However, in disease‐related prooxidant and inflammatory microenvironments, Apoa1 can be modified to alter the architecture of Apoa1 toward abnormalities and switch its protective properties to harmful properties.^[^ ^54^ ^]^

Our study demonstrated that Rpl17 regulates Apoa1 expression at both the mRNA and protein levels under E50K mutation, and we further identified the transcription factors of Apoa1. Among the candidate transcription factors, Stat5b was identified as a positive regulator of Apoa1. Interestingly, Stat5b protein levels were elevated in E50K microglia, despite no significant changes in Stat5b mRNA levels. Therefore, we hypothesized that Rpl17 may be involved in regulating the stability of Stat5b. Protein ubiquitination is one of the most common mechanisms affecting protein stability. We found that Rpl17 physically interacts and spatially colocalizes with Stat5b, which significantly inhibits Stat5b ubiquitination and contributes to the maintenance of its protein stability. These findings suggest that Rpl17 regulates the expression and secretion of Apoa1 in a Stat5b‐dependent manner, leading to increased Apoa1 in the retinal microenvironment, which in turn affects the viability of vascular endothelial cells. However, whether Apoa1 undergoes structural changes and the molecular mechanisms that lead to vascular endothelial cell damage remain to be elucidated and require further investigation in our subsequent experiments.

Given that Apoa1 is an extracellular protein that induces RVECs apoptosis, we asked whether Apoa1 can be targeted with drugs to reverse RVECs death and increase retinal peripapillary vascular density in NTG mice to protect RGCs and improve visual function. Through protein structure analysis and molecular docking screening, we found that EA can bind to Apoa1. EA is a naturally occurring polyphenolic compound that is abundant in various fruits, nuts, and plants.^[^ ^55^, ^56^, ^57^ ^]^ EA has beneficial effects on age‐related diseases such as diabetes, atherosclerosis and several types of cancer.^[^ ^25^, ^55^, ^58^, ^59^, ^60^, ^61^ ^]^ It has gained increasing attention in recent studies because of its safe antioxidant and anti‐inflammatory properties.^[^ ^62^, ^63^, ^64^ ^]^ However, studies on the role of EA in glaucomatous neuroprotection are still scarce.

Our study demonstrated that EA treatment reduced apoptosis in HUVECs cocultured with E50K_mut BV2 cells. Furthermore, blocking Apoa1 with EA increased retinal peripapillary vascular density compared with that before treatment and improved RGCs survival and visual function in both the L/D ‐ T and OMR tests, providing a new strategy for neuroprotection.

**Figure 8:**
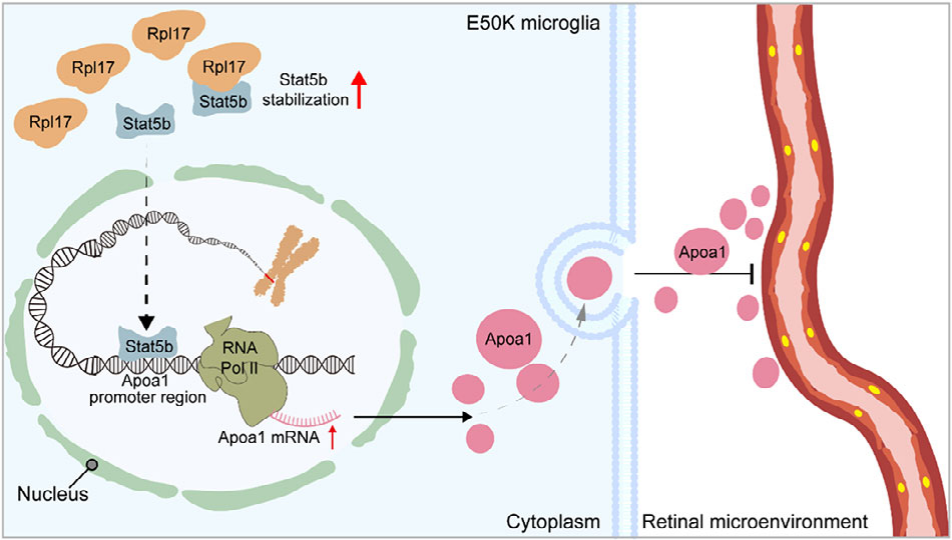
Diagram of the mechanisms described in this study.

In summary, we elucidated that E50K‐mutant microglia drive retinal peripapillary vascular density reduction in NTG via the Rpl17/Stat5b/Apoa1 axis and identified Apoa1 as a novel suppressor of retinal vascular endothelial cells (**Figure** **8**). These findings identify a potential therapeutic target for glaucoma and support the clinical potential of EA for protecting RGCs.

## 4 Experimental Section

### Cell Culture and Treatment

Human umbilical vein endothelial cells (HUVECs; HTX1922, Otwo Biotech, China) and murine microglia (BV2; AW‐CNM081, Abiowell, China) were used for in vitro studies. BV2 cells and HUVECs were cultured in 1640 medium supplemented with 5% fetal bovine serum (FBS) and 1% penicillin/streptomycin (Solarbio, China) at 37 °C with 5% CO_2_. The treatment was performed according to previously described procedures.^[^ ^65^ ^]^

### Animals

All experimental protocols were approved by the Institutional Animal Care and Use Committee at the Second Affiliated Hospital of Harbin Medical University and conformed to the Guide for the Care and Use of Laboratory Animals (NIH, 8th Edition, 2011) and the guidelines of the Ethics Committee of the Second Affiliated Hospital of Harbin Medical University (Permit Number: YJSDW2024‐155). OPTN (E50K) mice were generated via CRISPR‐Cas9 genome editing as an NTG mouse model, and age‐matched WT mice served as the control group. This NTG model mouse has been registered and named in the experimental animal database at The Jackson Laboratory in the United States (C57BL/6J‐Optn^em1Hyua^, MGI:7279070). The mice were housed in a specific pathogen‐free animal facility at the Second Affiliated Hospital of Harbin Medical University. Groups of mice with the OPTN (E50K) mutation at 3 and 16 months were designated the EY and EO groups, respectively, while the age‐matched WT control groups were correspondingly defined as the WY and WO groups. As previously described, EO mice presented more severe retinal degeneration than other mice did, similar to the degeneration observed in clinical NTG.^[^ ^8^ ^]^

### Visual Functional Analyses and Optical Coherence Tomography (OCT) Imaging

To examine the visual function of the mice, the L/D‐T and OMR tests were used, as previously described.^[^ ^7^, ^8^ ^]^ In brief, the L/D‐T test involves 2 equally sized chambers, a dark chamber and an illuminated chamber, with an aperture located in the middle wall allowing the mouse to move freely between them. After the mice were deprived of light for at least 2 h, a 10‐min test was performed, in which the length of time that the mice spent in the light chamber and the number of transitions between the 2 chambers were recorded. For the OMR test, LCD screens were placed around the inner wall to provide black and white stripes at different spatial rotation frequencies, while the mouse was placed at the platform in the center of the apparatus. Four spatial rotation frequencies were used: 0.05, 0.1, 0.2, and 0.3 cycles/degree (cpd). After adapting for a few minutes, the mice were tested at each frequency for 2 min, and head movements corresponding to the stripes were recorded.

For patient OCT imaging, the papillary and peripapillary VDs were generated by OCTA (Heidelberg Engineering, Heidelberg, Germany) with the Angio Disc (4.5 × 4.5 mm) mode. The RPC density and total vascular density in the RPC area were used for research and analysis. Mouse OCT imaging was performed at different retinal layers using Beiming‐Kun technology (400,000 ultrawideangle, full‐field, TowardPi Medical Technology Ltd., Beijing, China). The peripapillary vascular density in the ILM‐IPL and ILM‐OPL layers of the retina was used for research and analysis. Mouse eyes were routinely dilated after intraperitoneal anesthesia, according to the manufacturer's instructions, to capture high‐definition scans of different retinal layers.^[^ ^66^ ^]^

### Flat Mount of Retina

Retinal flat mounts were prepared to analyze vascular density, quantify RVECs, and count retinal RGCs. The mouse eyes were extracted and incubated with Triton X‐100 and 5% normal goat serum as described previously.^[^ ^8^ ^]^ Overnight incubation at 4 °C was performed with primary antibodies (Erg or NeuN) diluted in PBS. Alexa Fluor‐488 and Alexa Fluor‐594 were incubated as secondary antibodies for 2 h at room temperature. For vascular staining, IB4 (1:300; L2895, Merck, Germany) dye was added directly after permeabilization, followed by overnight incubation at 4 °C. The retinas were mounted with the vitreous side down on slides, and four radial cuts centered on the optic disc were made to flatten the tissue. The coverslips were incubated with antifade mounting medium (Dako Fluorescence Mounting Medium). Confocal fluorescence microscopy (Carl Zeiss, Celldiscoverer7, Germany) was used to capture tiled images, which were stitched into a mosaic covering the entire retinal flat mount. The vascular density around the optic disc was analyzed using the Angio tool. RVECs and RGCs counts were performed using ImageJ, with two rectangular regions (0.16 mm^2^, 400 × 400 µm) around the optic disc selected per retina, and all counts were conducted in a blinded manner.

### RNA Extraction and qRT‒PCR

RNA extraction and qPCR analysis were carried out following the same procedure as previously described.^[^ ^66^, ^67^ ^]^ RNA was extracted using TRIzol reagent (TaKaRa, Japan), and cDNAs were synthesized with a PrimeScript RT reagent kit from the same manufacturer. The SYBR PrimeScript RT‒PCR Kit (Roche, USA) was used to quantify the cDNA content according to the manufacturer's instructions. β‐actin served as an internal control. The 2^−△△Ct^ method was used to analyze the qRT‒PCR data. All primers were synthesized by Kumei Biotech, and detailed information is shown in Table S1 (Supporting Information).

### Protein Preparation and Western Blot

Western blotting was carried out following the same procedure as previously described.^[^ ^66^ ^]^ Total protein was extracted from cells and retinas using RIPA lysis buffer (P0013B; Beyotime, China). A total of 20 µg of each sample was diluted in sample loading buffer, denatured at 95 °C for 5 min, and electrophoresed on a 12.5% or 10% SDS polyacrylamide gel. Proteins were then transferred onto polyvinylidene fluoride (PVDF) membranes and blocked with 5% skim milk in Tris‐buffered saline containing 0.1% Tween‐20 (TBST) for 1 h at room temperature. The membranes were incubated overnight at 4 °C with the following primary antibodies: cleaved‐caspase3, Bax, Bcl2, Rpl17, Apoa1, and Stat5b. After three washes with TBST, the membranes were incubated with horseradish peroxidase‐conjugated secondary antibodies for 1 h at room temperature. The protein bands were visualized by enhanced chemiluminescence (ECL, MA0186, Meilunbio, China) and quantified with ImageJ. β‐actin served as the housekeeping protein. All antibody details are listed in Table S2 (Supporting Information).

### Immunofluorescence (IF)

IF was performed to visualize protein expression and localization in cells. Briefly, cells were seeded on cell slides (WHB‐24‐CS, Shanghai, China) in 24‐well plates and fixed using standard procedures after overnight incubation at 4 °C. The cells were incubated overnight at 4 °C with primary antibodies (Rpl17, Bcl2, Bax, cleaved‐caspase3, Apoa1, and Stat5b) diluted in PBS. Following three washes with PBS, the cells were incubated with secondary antibodies for 1 h at room temperature. The nuclei were stained with DAPI (C1002, Beyotime China), and images were captured using a confocal fluorescence microscope (Carl Zeiss, LSM 980, Germany).

### Cell Isolation by Flow Sorting

Flow cytometry was performed using a FACSCanto II flow cytometer and FACSAria III sorter (BD Immunocytometry Systems, San Jose, CA), and the results were analyzed using Flow Jo 9.6.4. Endothelial cells were sorted on the basis of CD45^–^CD31^+^ cells, and detailed information is shown in Table S3 (Supporting Information).

### Cell Transfection

Lentiviruses for OPTN knockout, OPTN (E50K) mutation, and Rpl17 knockdown, as well as a plasmid for Rpl17 overexpression, were constructed by Genechem (China). The sgRNA sequence used for OPTN knockout and the siRNA sequence used for Rpl17 knockdown are detailed in Table S4 (Supporting Information). The corresponding backbone vectors were used as controls. To achieve lentivirus‐mediated transduction, concentrated viral particles were added to culture medium containing polybrene (Sigma‒Aldrich, USA) and incubated with the cells for 24 h. The medium was subsequently replaced with complete culture medium, and successfully transduced cells were selected by culturing them for one week in the presence of the corresponding antibiotic for selection.

### Enzyme‐Linked Immunosorbent Assay (ELISA)

ELISAs were conducted on culture supernatants collected from BV2 cell cultures. Apoa1 levels were measured using ELISA kits (Ruifan, China) according to the manufacturer's instructions.

### Cell Apoptosis Analysis

For the cell apoptosis analysis, 1 × 10^6^ cells were resuspended to form a single‐cell suspension and washed twice with PBS. Apoptosis was assessed using an Annexin V‐FITC Apoptosis Detection Kit (BD Biosciences, USA) following the manufacturer's instructions. Apoptosis rates were determined by flow cytometry (BD FACSCanto II, USA).

### Statistical Analysis

Each experiment was conducted independently, with the sample size indicated in the corresponding figure legends. The data are presented as means ± standard deviations (SDs). Differences in variable groups were evaluated using Student's t test, Welch's t test or the Mann‒Whitney U test. The figures were generated using R version 4.3.0 with the pheatmap extension package. Significance was determined by *P* < 0.05. All the statistical analyses were performed using GraphPad software version 10.0 (GraphPad Software).

## Conflict of Interest

The authors declare no conflict of interest.

## Supporting information



Supplemental Figure

Supplemental Figure

Supplemental Figure

Supplemental Figure

Supplemental Figure

Supplemental Figure

Supplemental Figure

Supplemental Figure

Supplemental Figure

Supplemental Figure

Supplemental Figure

Supplemental Figure

Supplemental Table

Supplemental Table

Supplemental Table

Supplemental Table

## Data Availability

The data that support the findings of this study are available from the corresponding author upon reasonable request.
