# Supplementary figures and images for: Microglia Drive Peripapillary Vascular Density Reduction in Normal Tension Glaucoma by Regulating the Rpl17/Stat5b/Apoa1 Axis
Source: Adv Sci (Weinh). 2025 Sep 8;12(44):e07894. doi: 10.1002/advs.202507894 (PMC12667541; doi:10.1002/advs.202507894)

# Control

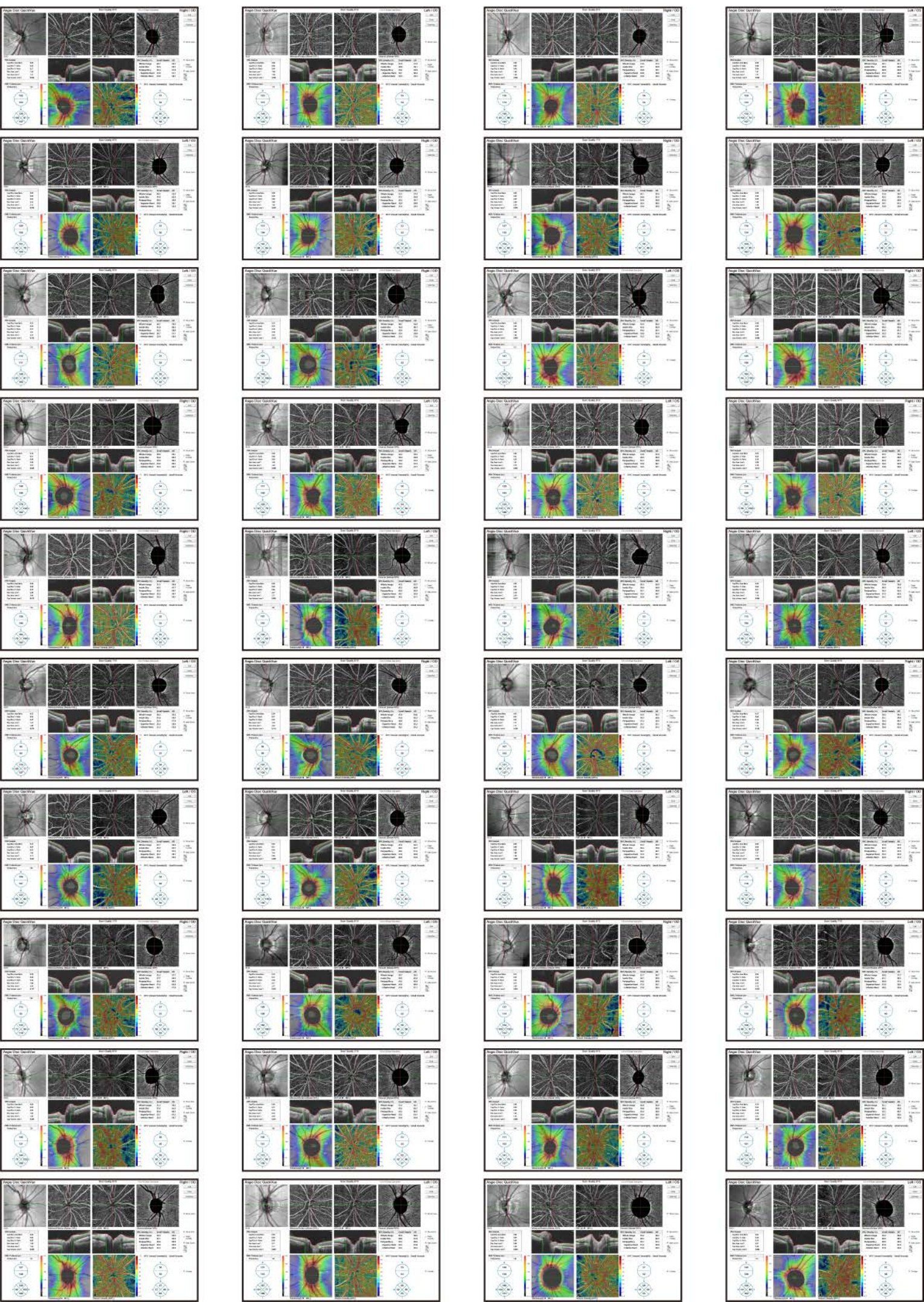

Supplement: Supplementary file 1 — Supplemental Figure [file ADVS-12-e07894-s012.pdf]

# NTG

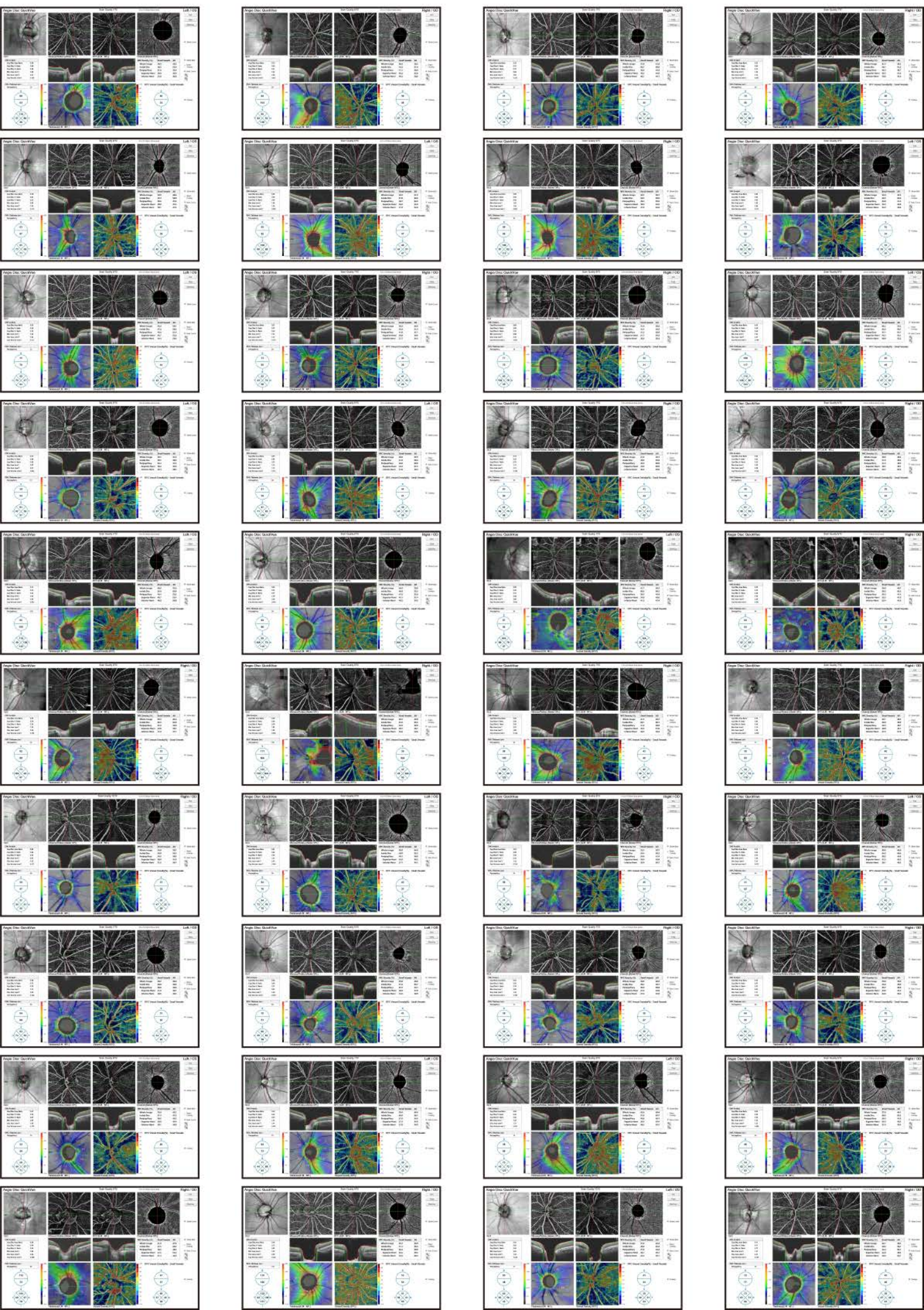

Supplement: Supplementary file 2 — Supplemental Figure [file ADVS-12-e07894-s011.pdf]

# WY ILM-IPL

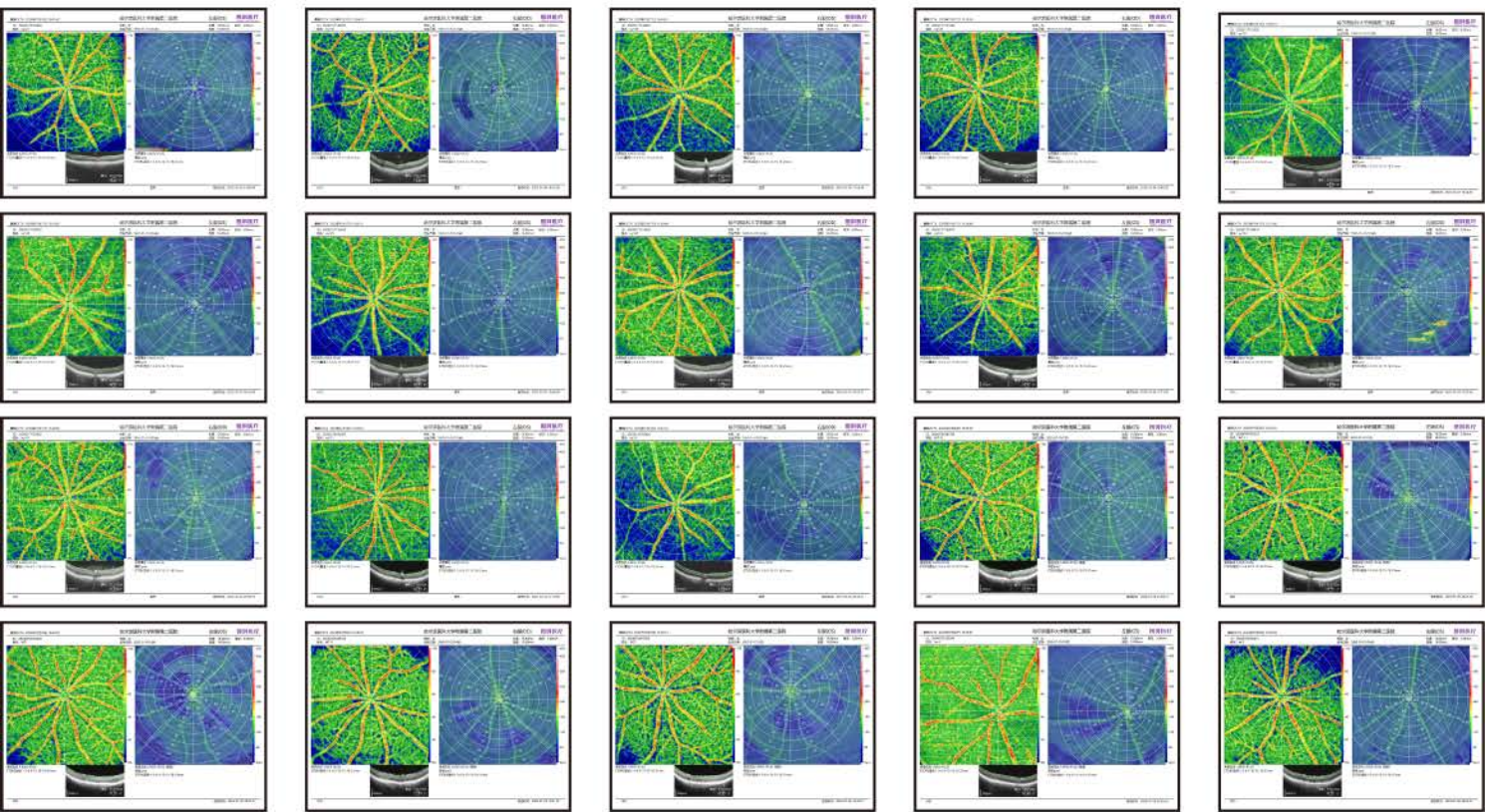

# EY ILM-IPL

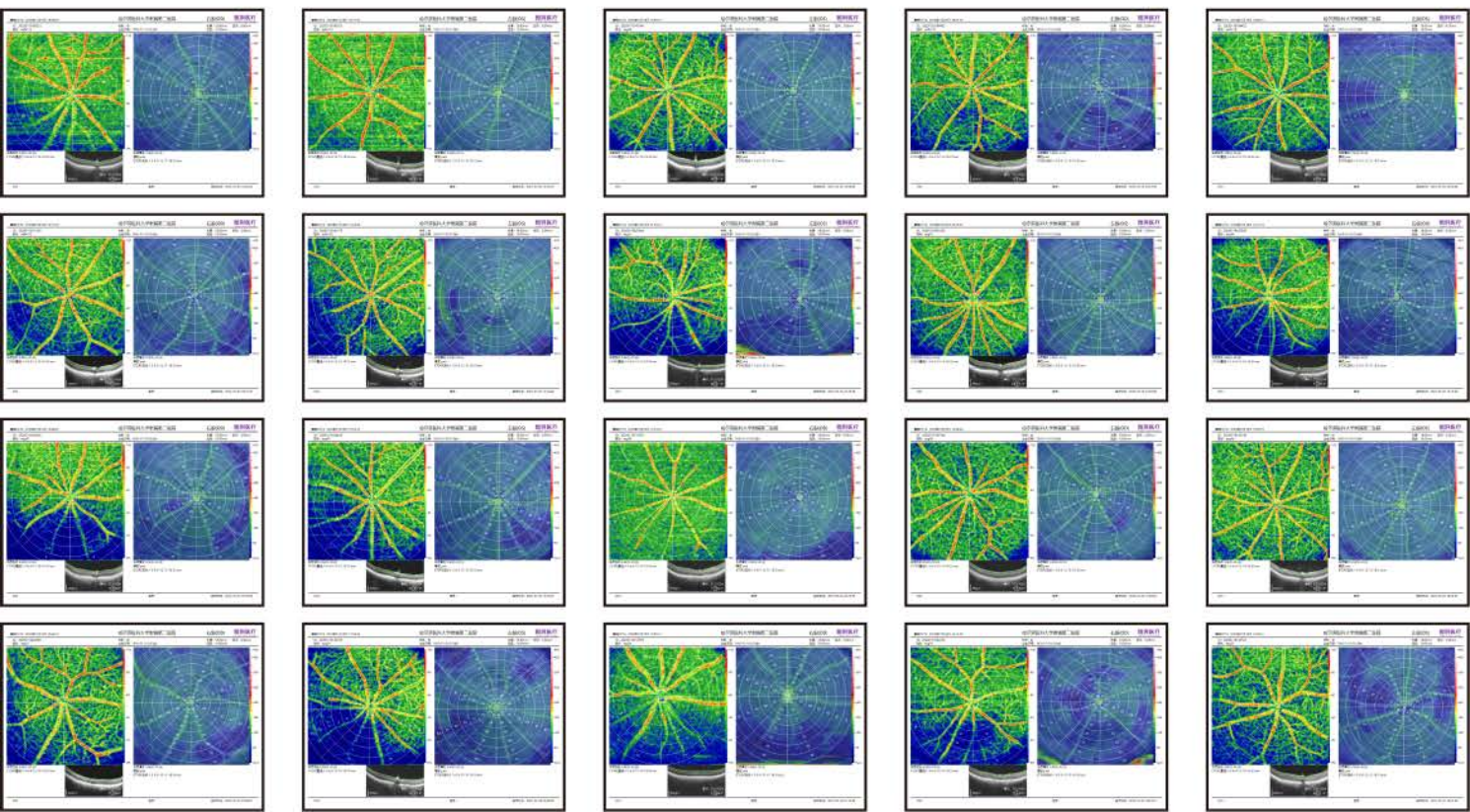

Supplement: Supplementary file 3 — Supplemental Figure [file ADVS-12-e07894-s008.pdf]

# WO ILM-IPL

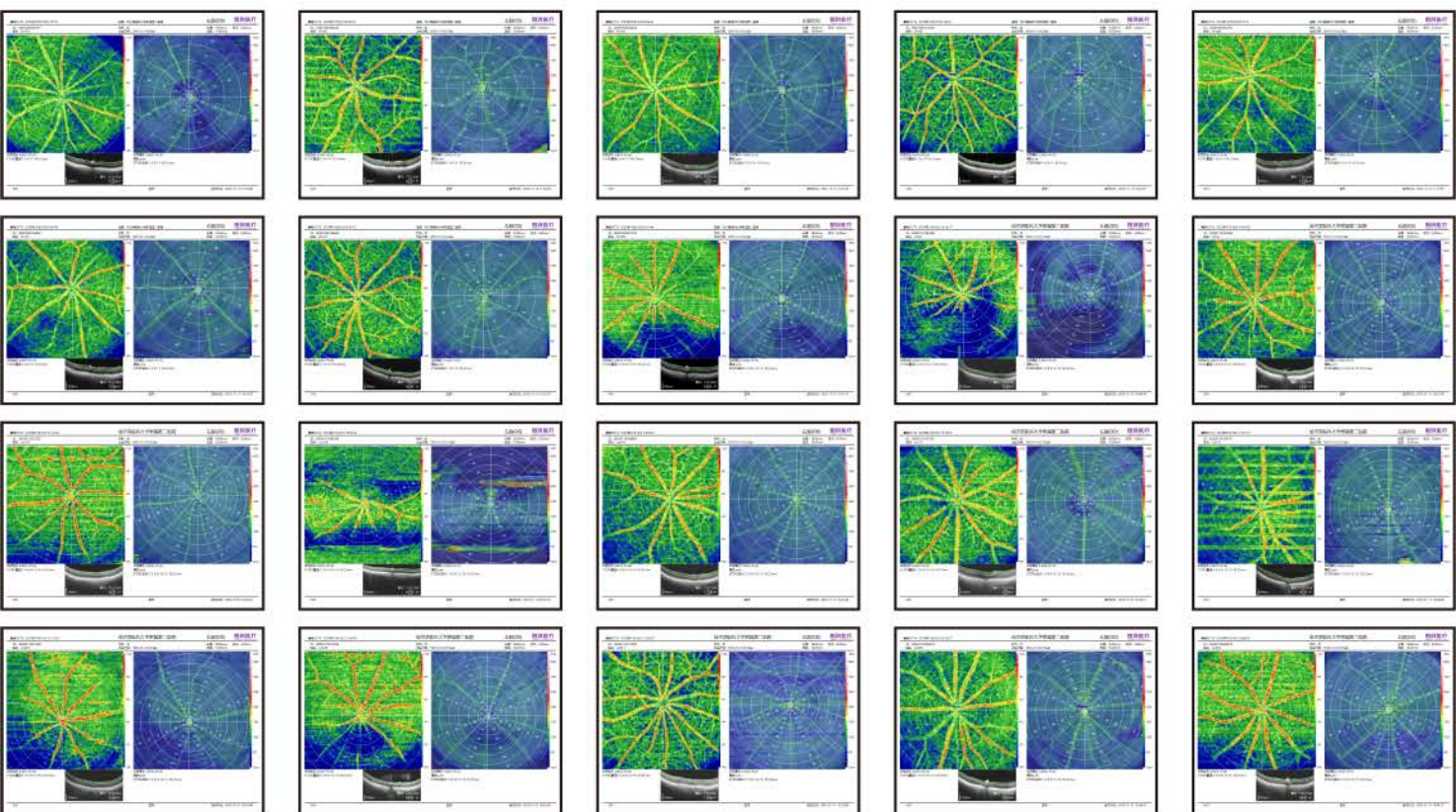

# EO ILM-IPL

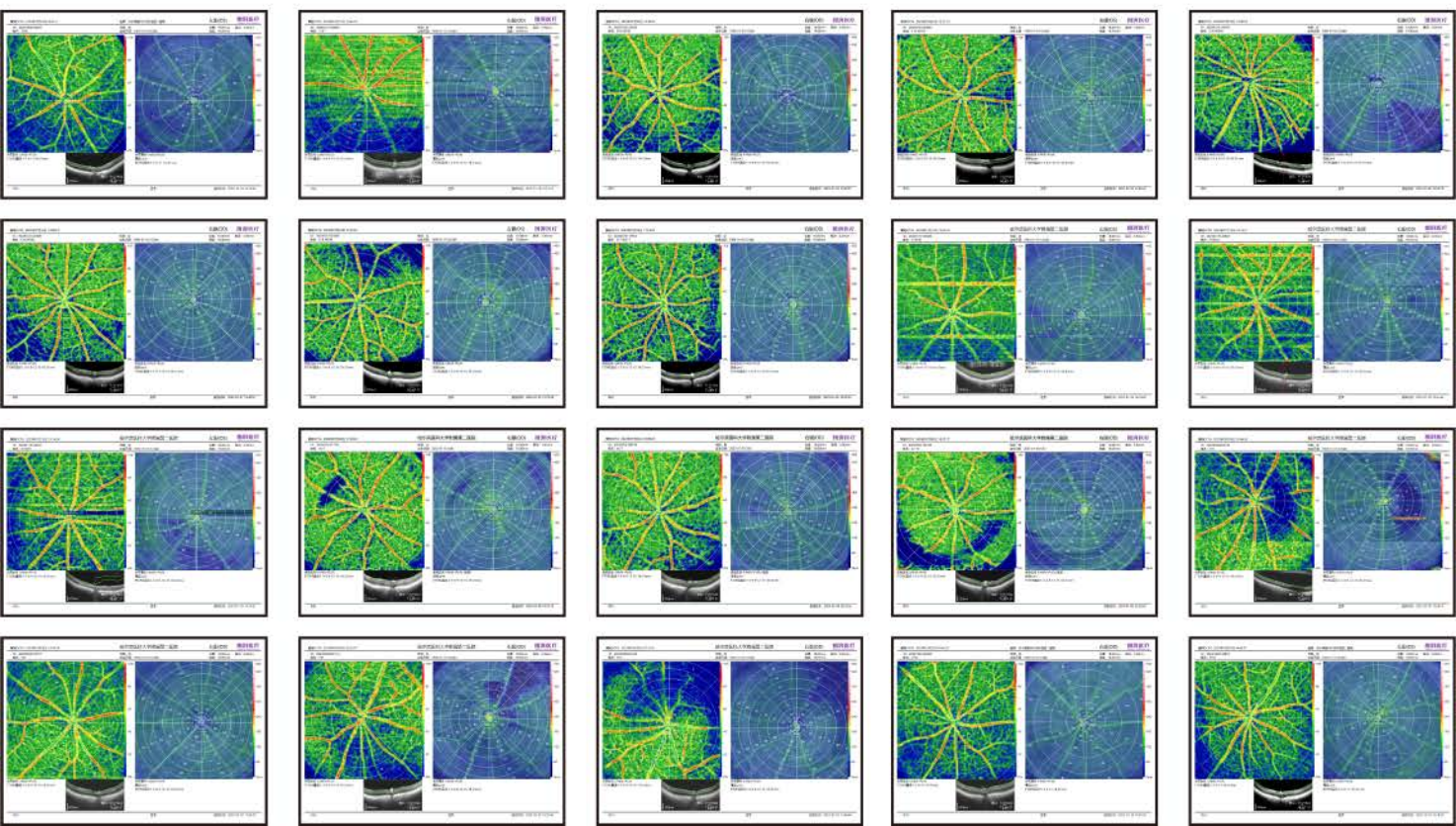

Supplement: Supplementary file 4 — Supplemental Figure [file ADVS-12-e07894-s009.pdf]

# WY ILM-OPL

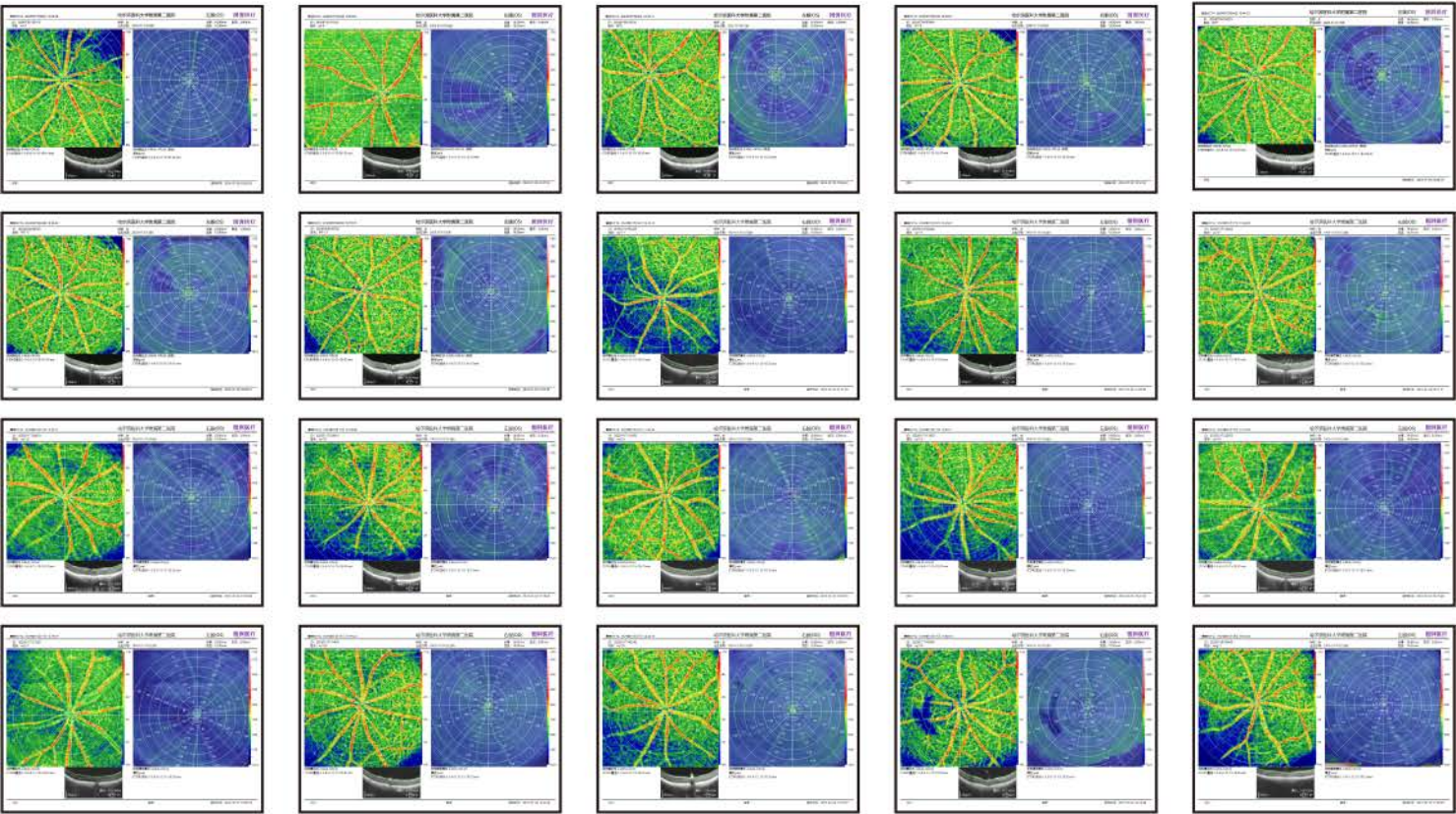

# EY ILM-OPL

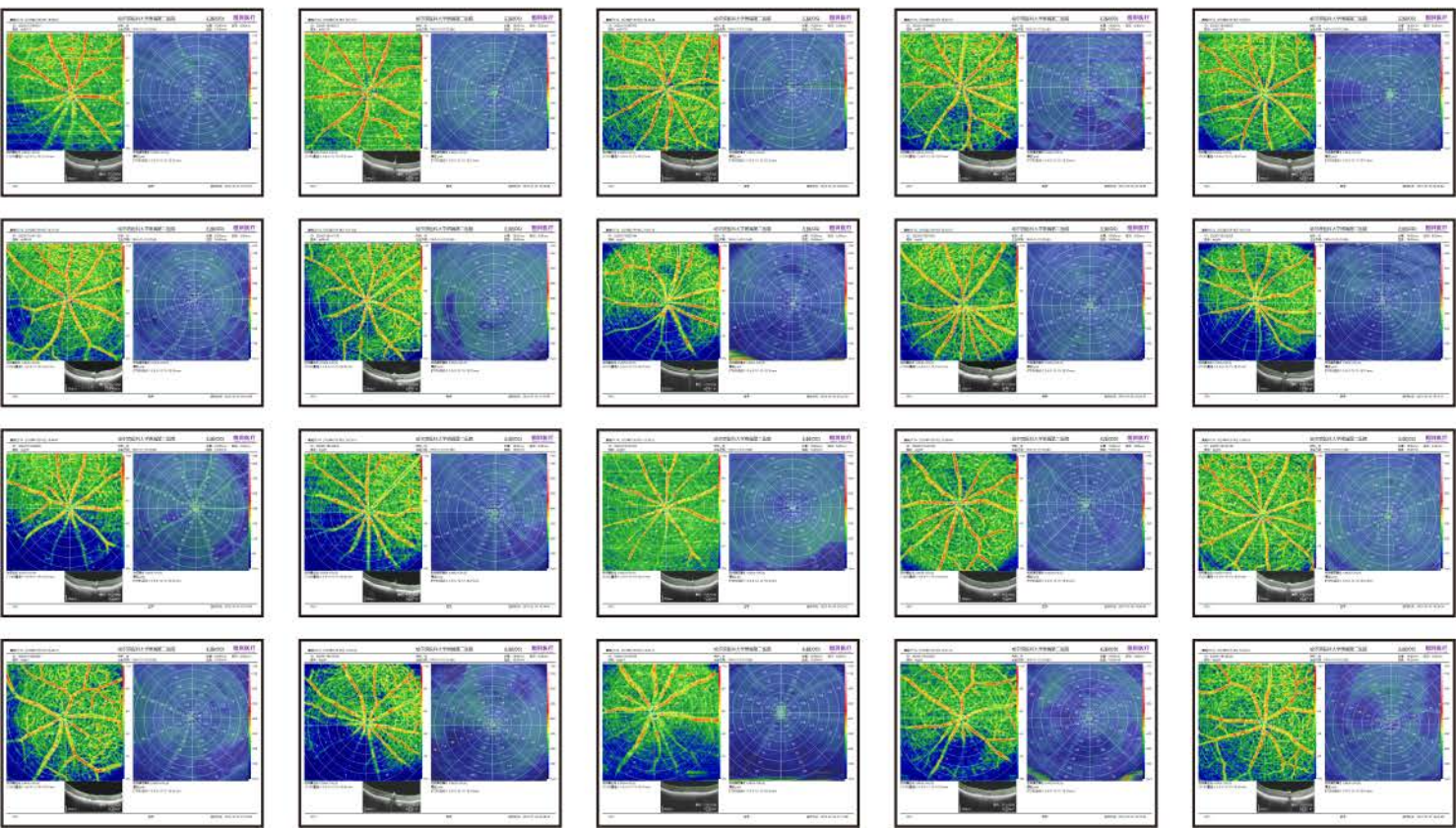

Supplement: Supplementary file 5 — Supplemental Figure [file ADVS-12-e07894-s006.pdf]

# WO ILM-OPL

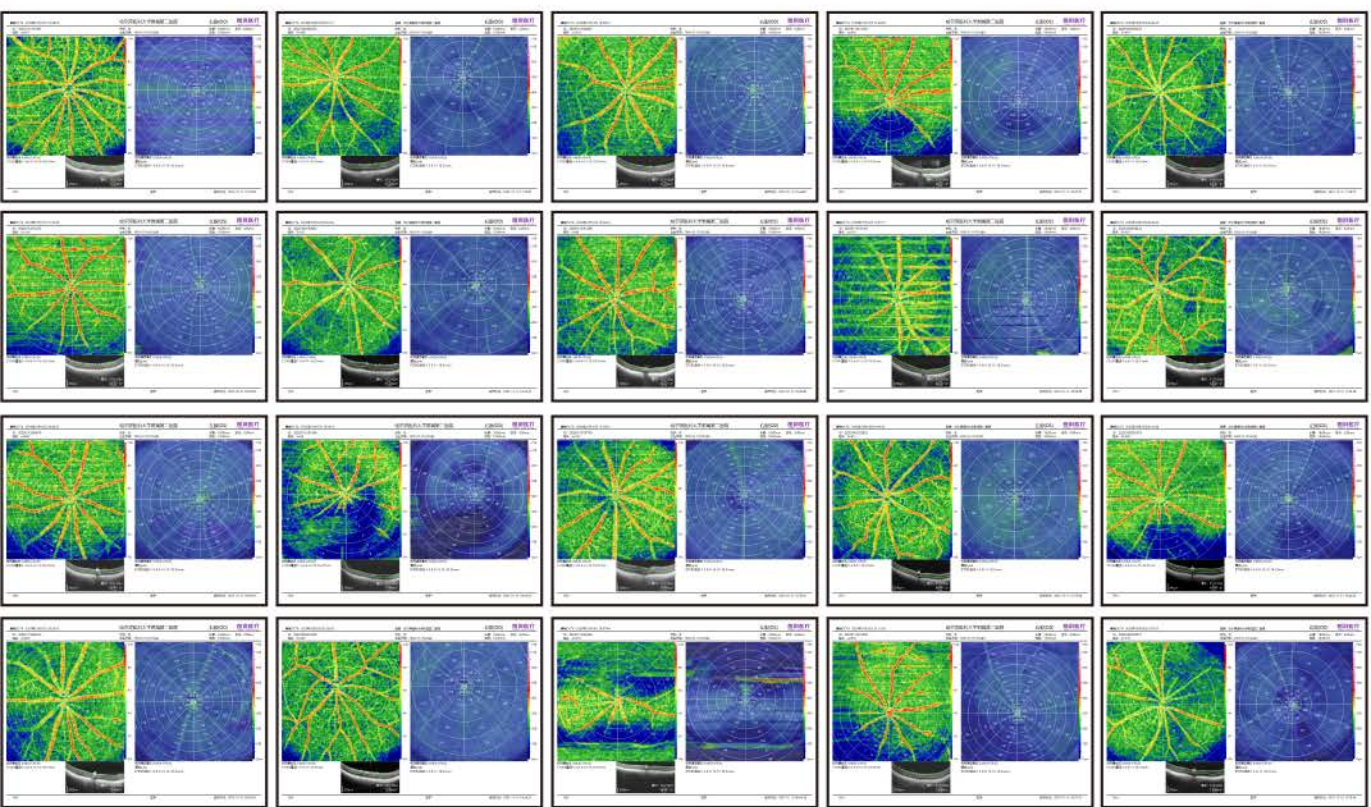

# EO ILM-OPL

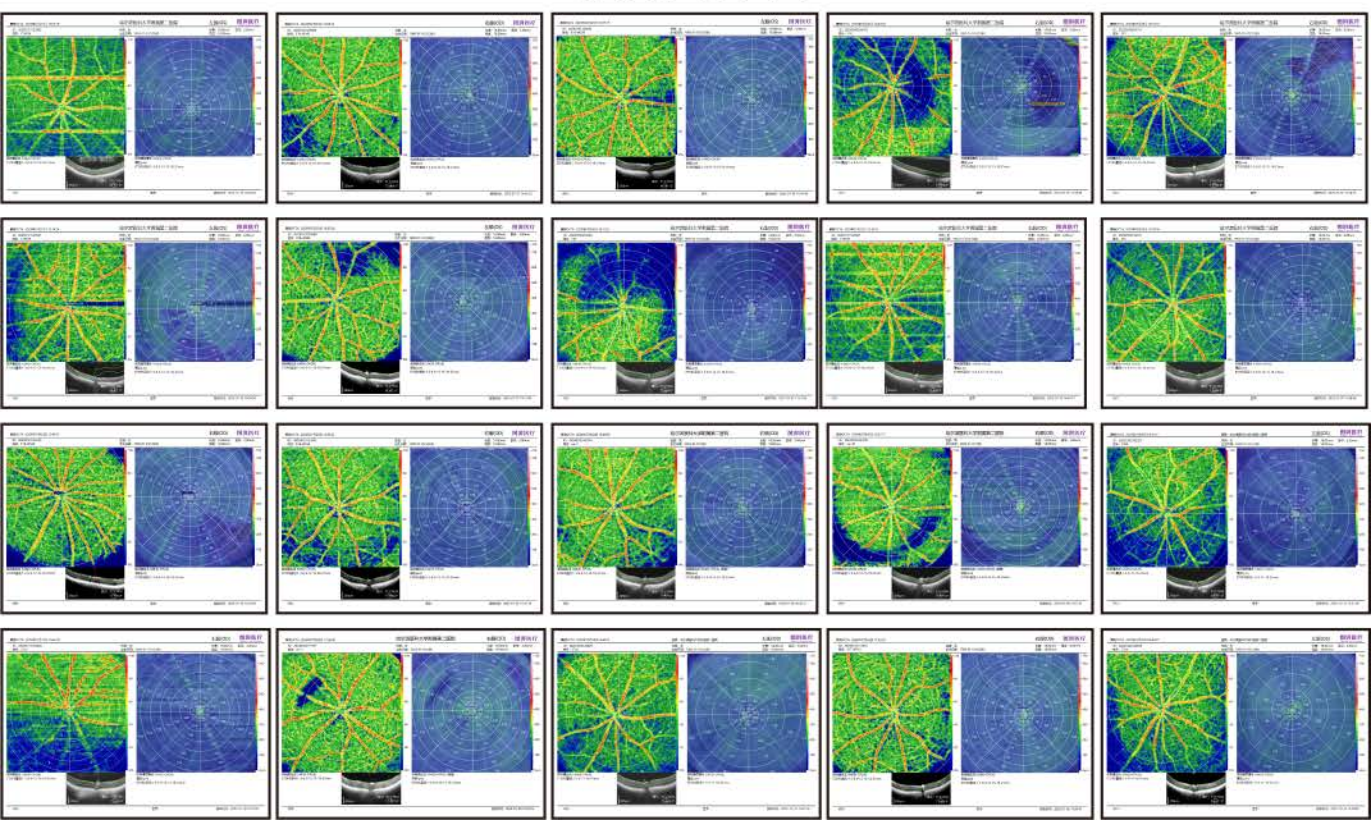

Supplement: Supplementary file 6 — Supplemental Figure [file ADVS-12-e07894-s013.pdf]

**A**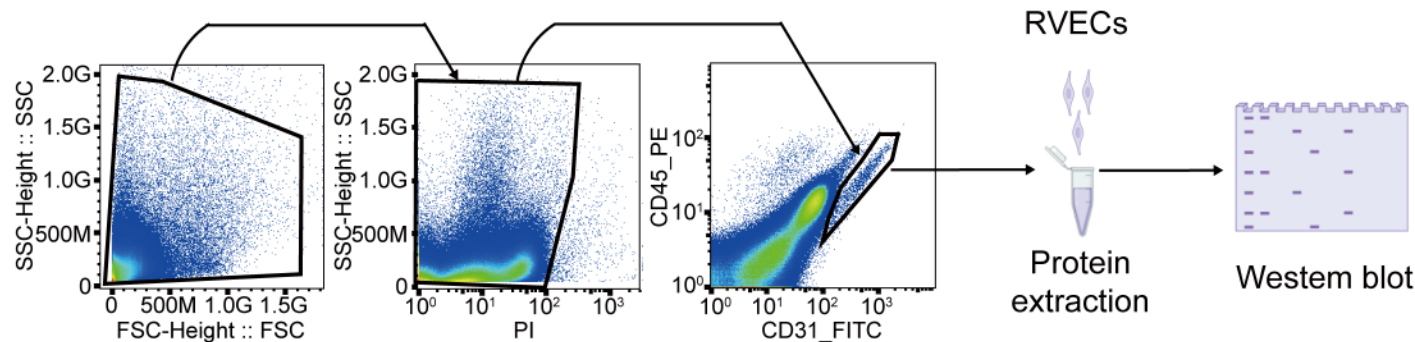**B**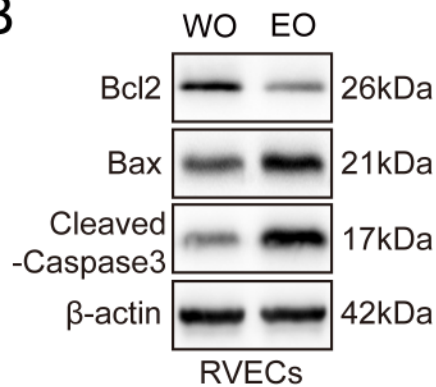**C**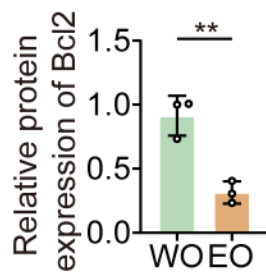**D**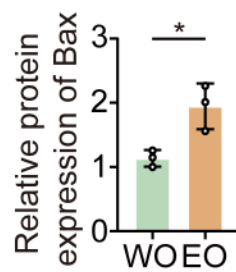**E**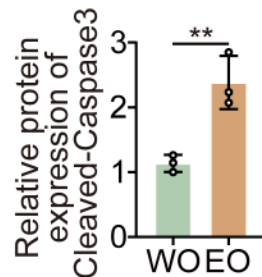**F**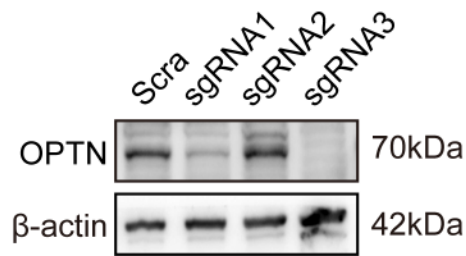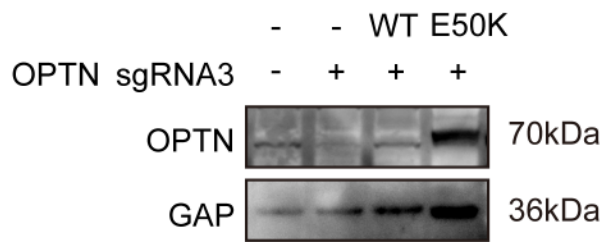

Supplement: Supplementary file 7 — Supplemental Figure [file ADVS-12-e07894-s002.pdf]

## Post-treatment

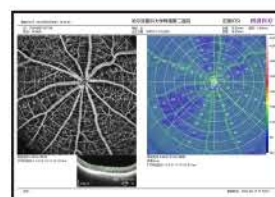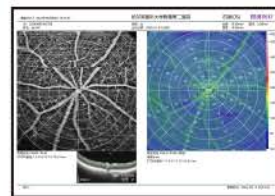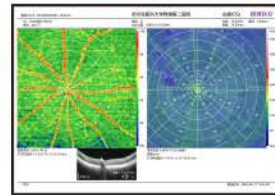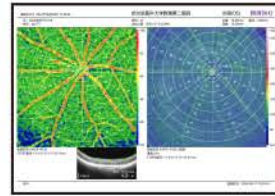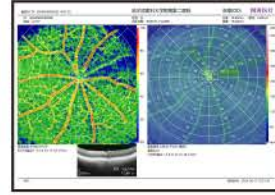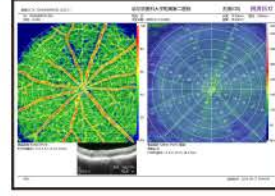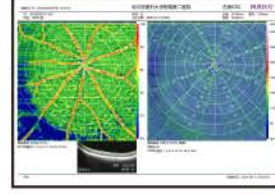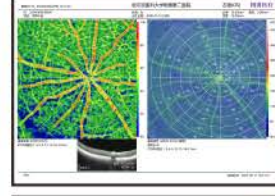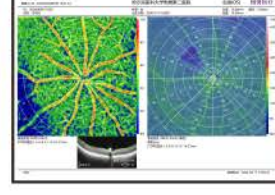

Supplement: Supplementary file 8 — Supplemental Figure [file ADVS-12-e07894-s004.pdf]

## Post-treatment

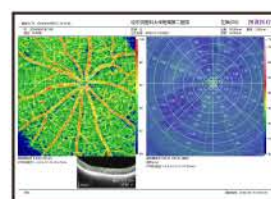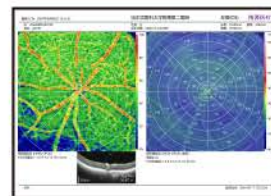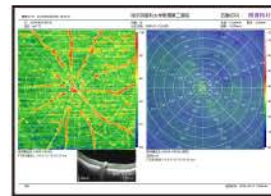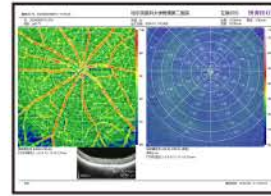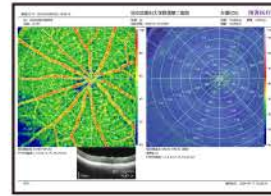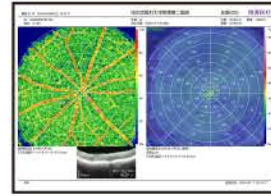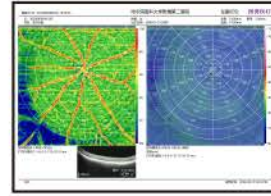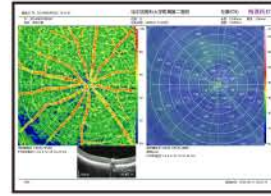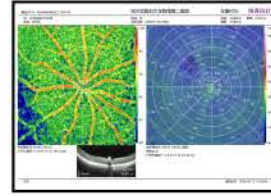

Supplement: Supplementary file 9 — Supplemental Figure [file ADVS-12-e07894-s016.pdf]

**A****EO+EA**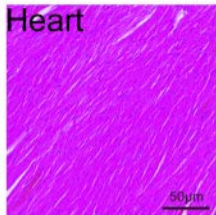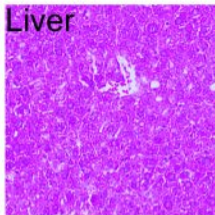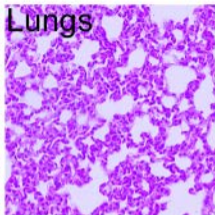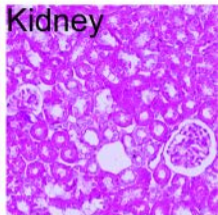**B****EO+EA**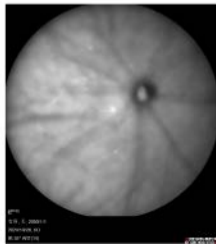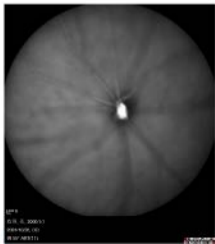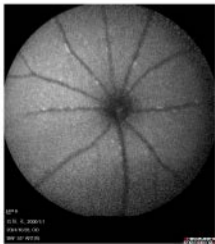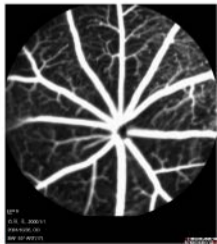

Supplement: Supplementary file 10 — Supplemental Figure [file ADVS-12-e07894-s010.pdf]

**Figure2F**

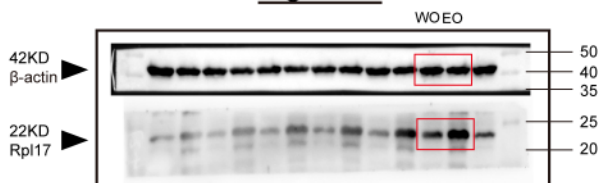

**Figure2K**

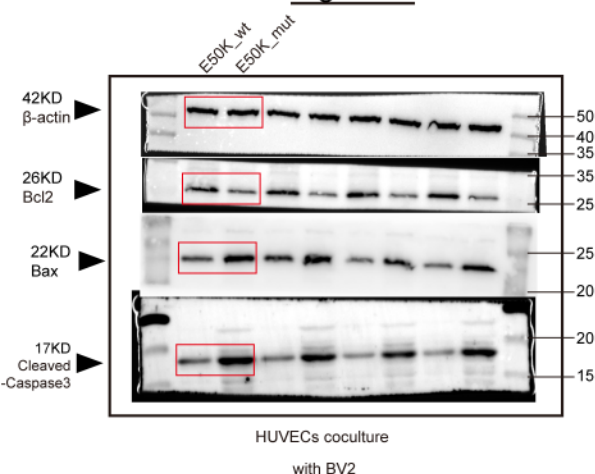

**Figure3N**

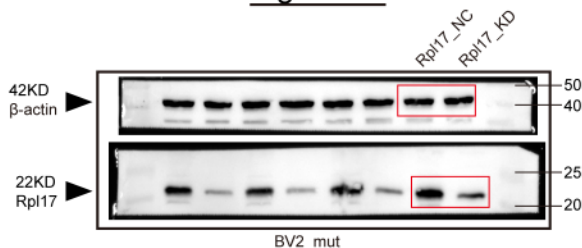

**Figure3V**

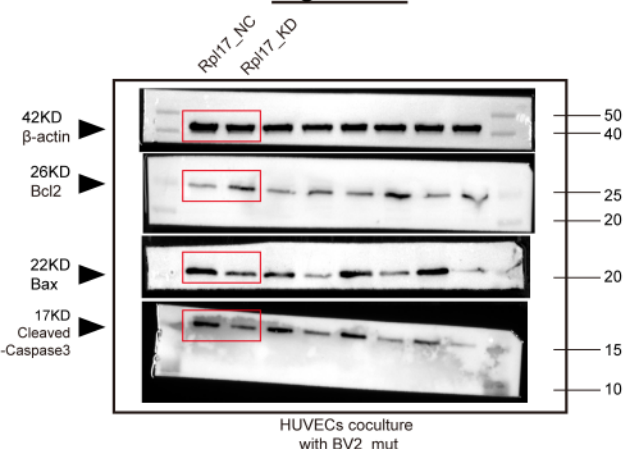

**Figure2H**

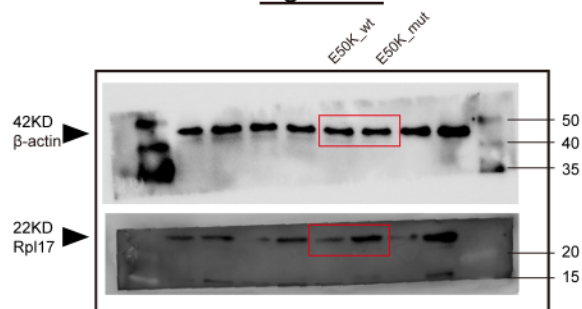

**Figure3E**

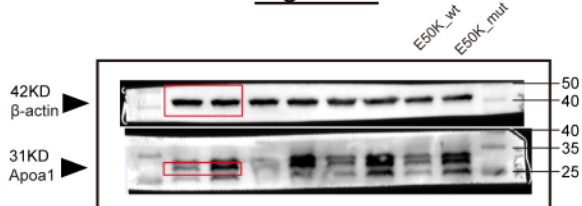

**Figure3J**

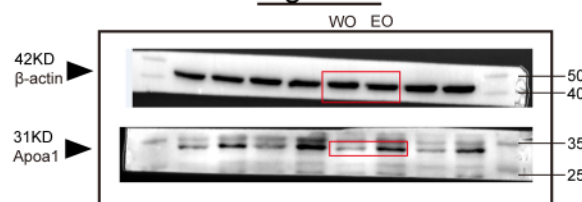

**Figure3R**

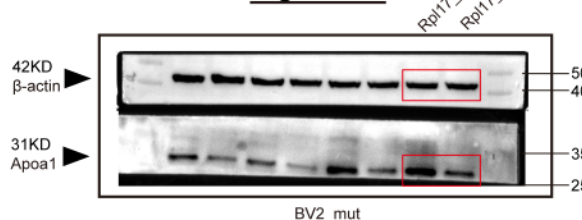

**Figure4E**

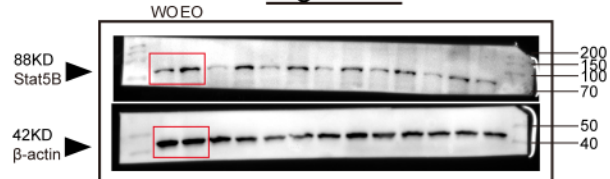

**Figure4H**

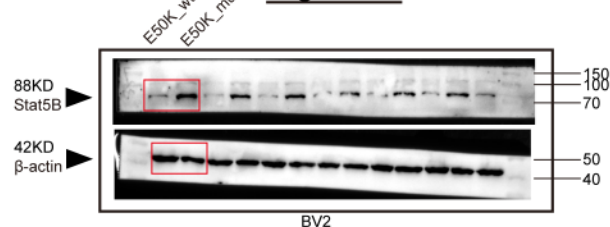

**Figure4K**

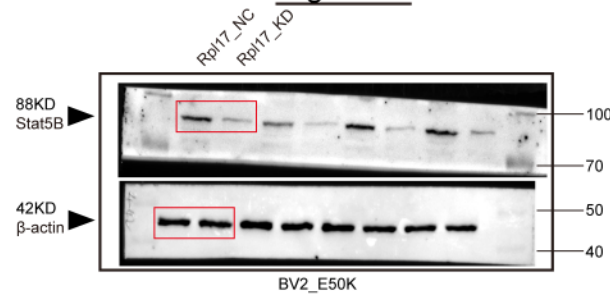

Supplement: Supplementary file 11 — Supplemental Figure [file ADVS-12-e07894-s007.pdf]

**Figure4P**

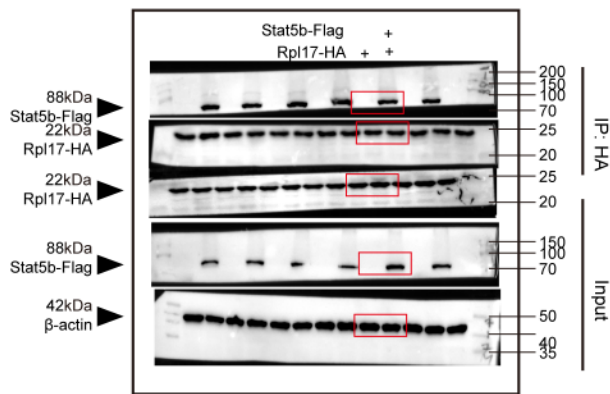

**Figure4Q**

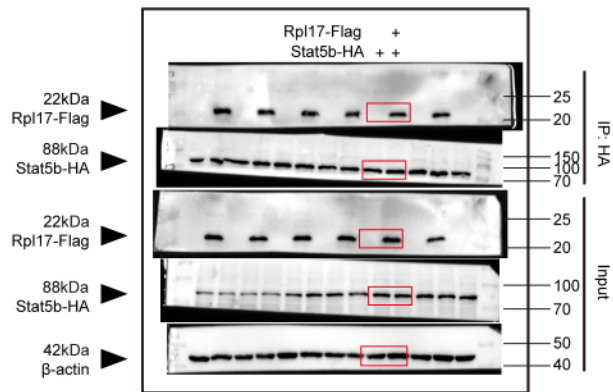

**Figure4T**

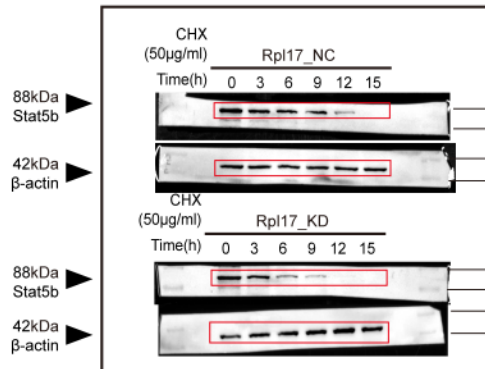

**Figure4U**

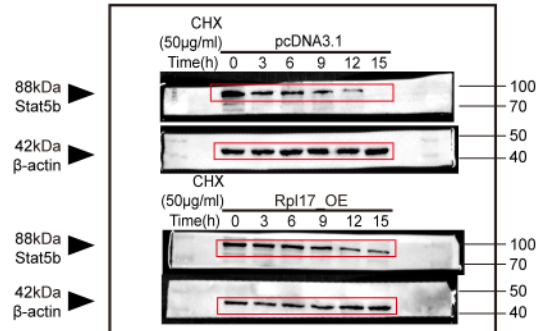

**Figure5G**

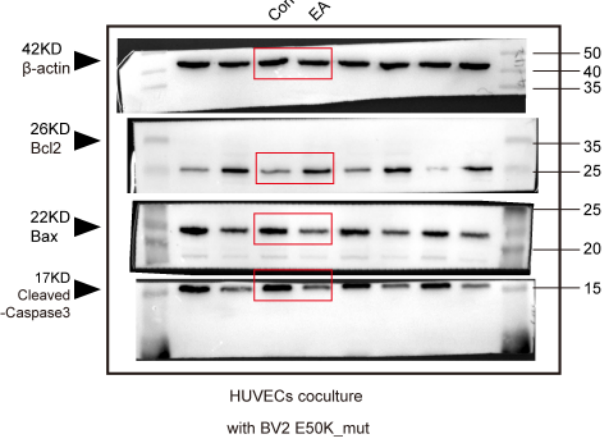

**Fig S7B**

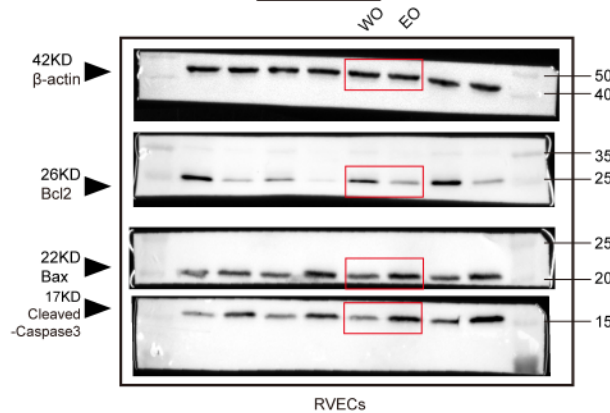

**Fig S7F**

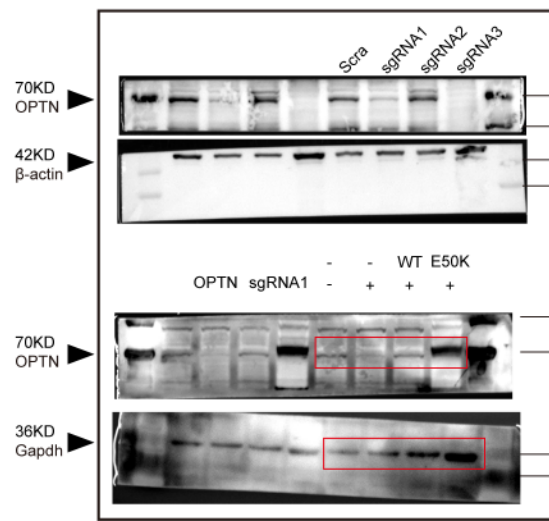

**Figure7A**

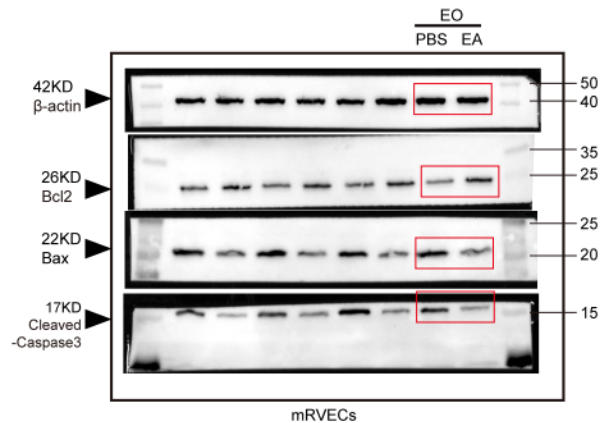

Supplement: Supplementary file 12 — Supplemental Figure [file ADVS-12-e07894-s005.pdf]
